# Creatine Supplementation Associated or Not with Strength Training upon Emotional and Cognitive Measures in Older Women: A Randomized Double-Blind Study

**DOI:** 10.1371/journal.pone.0076301

**Published:** 2013-10-03

**Authors:** Christiano Robles Rodrigues Alves, Carlos Alberto Abujabra Merege Filho, Fabiana Braga Benatti, Sonia Brucki, Rosa Maria R. Pereira, Ana Lucia de Sá Pinto, Fernanda Rodrigues Lima, Hamilton Roschel, Bruno Gualano

**Affiliations:** 1 School of Physical Education and Sport, University of Sao Paulo, São Paulo, Brazil; 2 School of Medicine, University of Sao Paulo, São Paulo, Brazil; National Institute of Agronomic Research, France

## Abstract

**Purpose:**

To assess the effects of creatine supplementation, associated or not with strength training, upon emotional and cognitive measures in older woman.

**Methods:**

This is a 24-week, parallel-group, double-blind, randomized, placebo-controlled trial. The individuals were randomly allocated into one of the following groups (n=14 each): 1) placebo, 2) creatine supplementation, 3) placebo associated with strength training or 4) creatine supplementation associated with strength training. According to their allocation, the participants were given creatine (4 x 5 g/d for 5 days followed by 5 g/d) or placebo (dextrose at the same dosage) and were strength trained or not. Cognitive function, assessed by a comprehensive battery of tests involving memory, selective attention, and inhibitory control, and emotional measures, assessed by the Geriatric Depression Scale, were evaluated at baseline, after 12 and 24 weeks of the intervention. Muscle strength and food intake were evaluated at baseline and after 24 weeks.

**Results:**

After the 24-week intervention, both training groups (ingesting creatine supplementation and placebo) had significant reductions on the Geriatric Depression Scale scores when compared with the non-trained placebo group (p = 0.001 and p = 0.01, respectively) and the non-trained creatine group (p < 0.001 for both comparison). However, no significant differences were observed between the non-trained placebo and creatine (p = 0.60) groups, or between the trained placebo and creatine groups (p = 0.83). Both trained groups, irrespective of creatine supplementation, had better muscle strength performance than the non-trained groups. Neither strength training nor creatine supplementation altered any parameter of cognitive performance. Food intake remained unchanged.

**Conclusion:**

Creatine supplementation did not promote any significant change in cognitive function and emotional parameters in apparently healthy older individuals. In addition, strength training *per* *se* improved emotional state and muscle strength, but not cognition, with no additive effects of creatine supplementation.

**Trial Registration:**

Clinicaltrials.gov NCT01164020

## Introduction

Aging has been associated with cognitive impairment and depressive symptoms [1–3], which, in turn, may lead to emotional and social isolation, and, hence, poor quality of life [4,5]. The current pharmacological tools for counteracting both depression and cognitive decline have limited efficacy and are not free of adverse effects. Consequently, novel non-pharmacological strategies to prevent mental disorders secondary to aging have been encouraged. In this regard, creatine supplementation and strength training have emerged as promising tools able to improve mental health in older individuals.

Creatine (*N*-aminoiminomethyl-*N*-methylglycine) is a guanidine compound synthesized by kidneys, pancreas, and liver (approximately 1 g/d), as well as ingested from food (approximately 1-5 g/d). Creatine may bond to a phosphoryl group to form phosphorylcreatine. Creatine kinase (CK) catalyzes the reversible transfer of the *N*-phosphoryl group from phosphorylcreatine to adenosine diphosphate (ADP) to regenerate adenosine triphosphate (ATP) [6].

There is high-quality evidence showing that creatine exerts a vital role in cerebral energetic provision, corroborated by i) the presence of creatine kinase (PCK) isoforms in both the brain and spinal cord [7]; ii) the association between brain creatine depletion and mental retardation, autism, speech delay, and brain atrophy [8]; and iii) the reversal of these symptoms following oral creatine administration [9]. Recently, it has been suggested that orally ingested creatine can penetrate the blood–brain barrier, thereby improving brain energy metabolism in humans [9–12]. Interestingly, it was reported that creatine supplementation may also alleviate mental fatigue induced by stressor stimulus, such as mathematical calculus [13] and sleep deprivation combined with vigorous physical activity [14]. Furthermore, there is evidence demonstrating that creatine supplementation can improve mood in patients with treatment-resistant depression [15] or post-traumatic stress disorders associated or not with comorbid depression [16], possibly restoring their brain creatine levels, which has been proven to be reduced in psychiatric illnesses [17,18,19,20,21,22). In light of this body of knowledge, one could speculate that creatine supplementation could improve cognition and emotional state in older individuals.

Physical fitness and physical activity levels have been inversely associated with cognitive decline and dementia [2,23–28]. For instance, in a recent meta-analysis of prospective studies involving 33816 individuals, mild to moderate physical activity levels were associated with a reduction of 35% of cognitive impairment [29]. Interestingly, some studies have also found a positive role of physical fitness on depression [4,5]. Even though the majority of the studies assessing the influence of training in cognitive function and depression have employed aerobic-type physical activities, recent studies have also demonstrated a potential role of strength training at improving depression and cognitive performance in elderly people [4,30–32]. For instance, Perrig-Chiello et al. (1998) showed that a one-session-a-week strength training program for 8 weeks produced modest effects on selected cognitive tasks (i.e., free recall and recognition) in elderly population. In support of these findings, Cassilhas et al. (2007) found improvements in several cognitive parameters (i.e. short-term memory, long-term episodic memory, and attention) after a 24-week strength training program performed three times a week at two different intensities (moderate: 50% of one RM and high intensity: 80% of one RM). Moreover, Liu-Ambrose et al. (2010) demonstrated that a strength training program performed once or twice a week equally improved the selective attention and the susceptibility to interference from conflicting stimuli, as assessed by the Stroop Test. Cassilhas et al., (2010) also reported the efficacy of high-intensity strength training at improving mood and anxiety in older subjects. Altogether, these data indicate that strength training may be a useful strategy to improve cognitive performance and emotional measures in old populations.

Recently, the combination of creatine supplementation and strength training has emerged as an efficient non-pharmacological tool in counteracting some aspects of sarcopenia, including physical dysfunction, disability in activities of daily living, low lean mass, and poor quality of life (for a comprehensive review, see 33). Nonetheless, the influence of this strategy upon cognitive and emotional measures, although promising, remains scarcely examined by randomized controlled trials.

In spite of the potential therapeutic role of isolated strength training or creatine supplementation on cognitive function and emotional state in older individuals, no studies have investigated the possible additive effects of these strategies combined. Therefore, the aim of this study was to assess the effects of creatine supplementation, associated or not with strength training, upon cognitive and emotional measures in older individuals.

## Material and Methods

### Experimental protocol and sample

The protocol for this trial and supporting CONSORT checklist are available as supporting information; see Checklist S1, Protocol S1, and Protocol S2 (Portuguese).

This was a 24-week, parallel-group, double-blind, centrally randomized, placebo-controlled trial conducted between February 2011 and December 2012 at São Paulo, Brazil. This study was registered at clinicaltrials.gov as NCT01164020. The protocol was approved by the local Ethics Committee (General Hospital, School of Medicine, University of Sao Paulo). All of the participants were fully informed of the risks and discomforts associated with the study before giving their written informed consent. All of the procedures were in accordance with the Helsinki Declaration revised in 2008. This manuscript is reported according to the CONSORT statement.

The sample was composed by 56 healthy older women aged 66.8 years (range of 60 to 80 years). Before entering the study, the participants were submitted to a medical examination and a maximal ergometric test to determine eligibility. The exclusion criteria were as follows: i) cardiovascular involvement (e.g., arrhythmias, arterial hypertension, heart failure, myocarditis, and pericarditis); ii) current tobacco usage; iii) previous creatine supplements usage; and iv) other chronic diseases (e.g., diabetes mellitus, rheumatoid arthritis, chronic kidney disease, hepatic diseases or psychiatric comorbidity, including clinically diagnosed depression). None of the participants were engaged in any regular physical fitness program for at least one year prior to the study.

**Figure 1:**
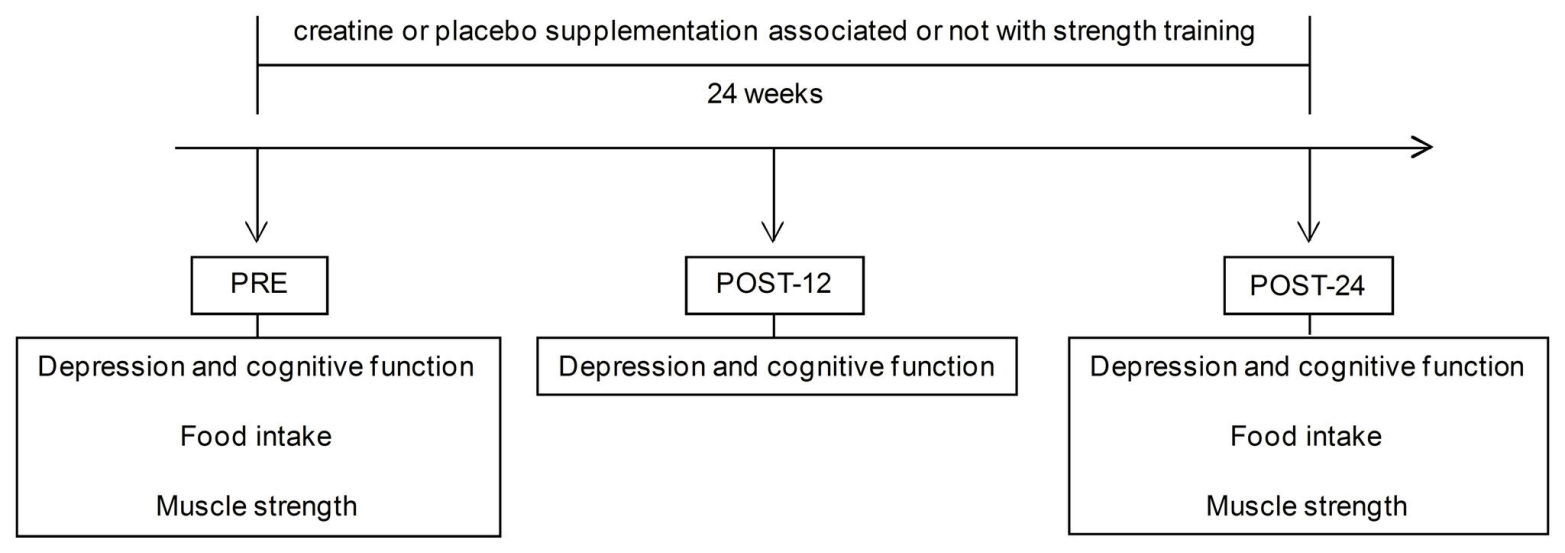
Experimental design.

The participants were randomized using a computer-generated randomization code (Minitab v.15) in blocks of eight in a 1:1:1:1 ratio to compose either one of the following groups: 1) placebo supplementation (PL; n = 14), 2) creatine supplementation (CR; n = 14), 3) placebo supplementation associated with strength training (PL+ST; n = 14) or 4) creatine supplementation associated with strength training (CR+ST; n = 14). Emotional and cognitive parameters (the primary outcomes) were assessed at baseline (PRE), after 12 weeks (POST-12) and after 24 weeks (POST-24) of creatine supplementation and/or strength training. Muscle strength (the secondary outcome) and food intake were assessed only at PRE and POST-24. Moreover, adverse events were recorded throughout the trial. Figure 1 illustrates the experimental design.

Evaluations of muscle function (by timed-stands test and timed-up-and-go test) and body composition (by dual energy X-ray absorptiometry) were originally planned, but not performed due to technical issues. In addition, after a pilot-study, we added some questionnaires (including a specific one to assess emotional state; see below) to the battery of cognitive tests originally designed.

### Supplementation protocol

The CR and CR+ST groups received 20 g of creatine monohydrate (4 x 5 g/d) for five days followed by 5 g/d as a single dose throughout the trial. The PL and PL+ST groups were given the same dose of dextrose. The individuals were advised to consume their supplements preferably along with meals (e.g., breakfast, lunch, afternoon snack, and dinner). The supplement packages were coded so that neither the investigators nor the participants were aware of the contents until completion of the analyses. The supplements were provided by a staff member of our research team who had not any participation in the data acquisition, analyses, and interpretation. In order to verify the purity of the creatine used, a sample was analyzed by HPLC and purity was established as 99.9%.

### Strength training protocol

The strength training was performed in an intrahospital gymnasium (School of Medicine, University of Sao Paulo) and consisted of twice a week sessions of supervised strength training for 24 weeks. Each session lasted approximately 40 minutes and consisted of 3 sets of 12-15 maximum repetitions (RM) for 7 exercises (i.e., chest press, leg press, lat pull-down, leg extension, rowing, squat, and sit ups), with 1-min interval between sets. The progression of the training load for each exercise was implemented when the subjects were able to properly perform more than 15-RM in two consecutives training sessions.

### Assessments of muscle strength

In order to assess the efficacy of the strength training in increasing muscle strength, the participants were submitted to 1-RM tests. Prior to the actual tests, three familiarization sessions were performed, separated at least 72 h from each other. Prior to the 1-RM tests, two warm-up sets interspaced by two-minute intervals were performed. Thereafter, the patients had up to five attempts to achieve the 1-RM load (e.g., maximum weight that could be lifted once with proper technique), with a three-minute interval between attempts. 1-RM tests were conducted for the chest-press and leg-press exercises.

### Food intake assessment

Food intake was assessed by three 24-h dietary recalls undertaken on separate days (two week days and one weekend day) using a visual aid photo album of real foods. The 24-h dietary recall consists of listing the foods and the beverages consumed during 24h prior to the recall. Energy and macronutrient intakes were analyzed by the Brazilian software Virtual Nutri^®^.

### Emotional and cognition measures

The application of the battery of cognitive and emotional tests lasted no more than 30 min. The tests were applied individually by the same experienced examiner during the morning (7:00 to 11:00 a.m.) in an appropriate office. Emotional state was assessed by the shorter version of the Geriatric Depression Scale. Cognitive function was assessed by a battery of tests comprising the Mini-Mental State Examination, Stroop Test, Trail Making Test, Digit Span Test, and Delay Recall Test.

The shorter version of the Geriatric Depression Scale is a self rating scale, including 15 items for dysphoria [34]. It has been tested and used extensively with the older population. It is a brief questionnaire in which participants are asked to respond to the 15 questions by answering “yes” or “no” in reference to how they felt on the day of administration. Higher scores indicate more "depressive state". It is worth noting that this scale was used exclusively to compare emotional state at baseline with the post-test scores rather than diagnose depression. Mini-Mental State Examination has been used as a brief neuropsychological screening for cognitive impairment consisting of questions on temporal and spatial orientation, memory, attention/concentration, and language and constructional praxis. Higher scores mean better performance in the test [35,36]. The Stroop Test (Victoria version) has been considered as a measure of the selective attention, susceptibility to interference from conflicting stimuli, and inhibitory control [37]. It includes three conditions that consist in naming the color of dots (i.e., “color”), neutral words (i.e., “non-color word”), and color words printed in incongruent colors (i.e., “color word”). Performance is assessed based on the time to complete each condition. The Trail Making Test has been used to assess executive functions and motor speed. It includes two conditions (i.e., “A” and “B”), where condition “A” reflects both motor and visual control and condition “B” condition reflects the additional executive control needed to switch between number and letter sequences [38]. Performance is assessed based on the time to complete each condition. However, only a small part of the participants (n = 25) was able to complete the condition “B” at baseline, so that only Part “A” was considered in the analysis. In order to assess the short-term memory, we applied the Digit Span Test [39], which requires the participant to orally repeat a sequence of digits forwards and backwards. The performance is assessed based on the number of digits that the participant is able to correctly recall. We also used the Delayed Recall Test of the Brief Cognitive Screening Battery [40], which consists of 10 line-drawings that are presented three times to the participant, then five minutes later, the subject is asked to recall as many drawings as possible.

All the above described instruments were previously validated to Brazilian population.

### Statistical analysis and post-hoc power analysis

To mitigate the impact of inter-individual data variability, all values were converted into delta scores (i.e., POST – PRE values) and thereafter tested by a mixed-model assuming “pre values” as a covariate. We used the approximate inference about fixed effects in mixed linear models (i.e., Kenward-Roger correction) in order to deal with the unbalanced design. Tukey *pos-hoc* was used for multicomparison purposes. Fisher’s test was applied to assess the efficacy of the blinding procedure. Data are presented as mean ± standard deviation and 95% confidence interval (95% CI), except when otherwise stated. The significance level was previously set at p < 0.05.

We calculated the achieved power (1 -β error probability) of the analysis with the assistance of the G-Power^®^ software (version 3.1.2). The effect size for the Geriatric Depression Scale scores (one of the primary outcomes) in the CR+ST group and the PL+ST group (-0.81 and -0.59) were inputted in the analysis with an α error probability of 0.05. The total sample size (n = 47), the numerator degree of freedom (3), the number of groups (4) and the number of covariates (1) were also used in the equation. The output for the achieved power estimation ranged from 0.99 to 0.91.

## Results

### Sample

**Figure 2:**
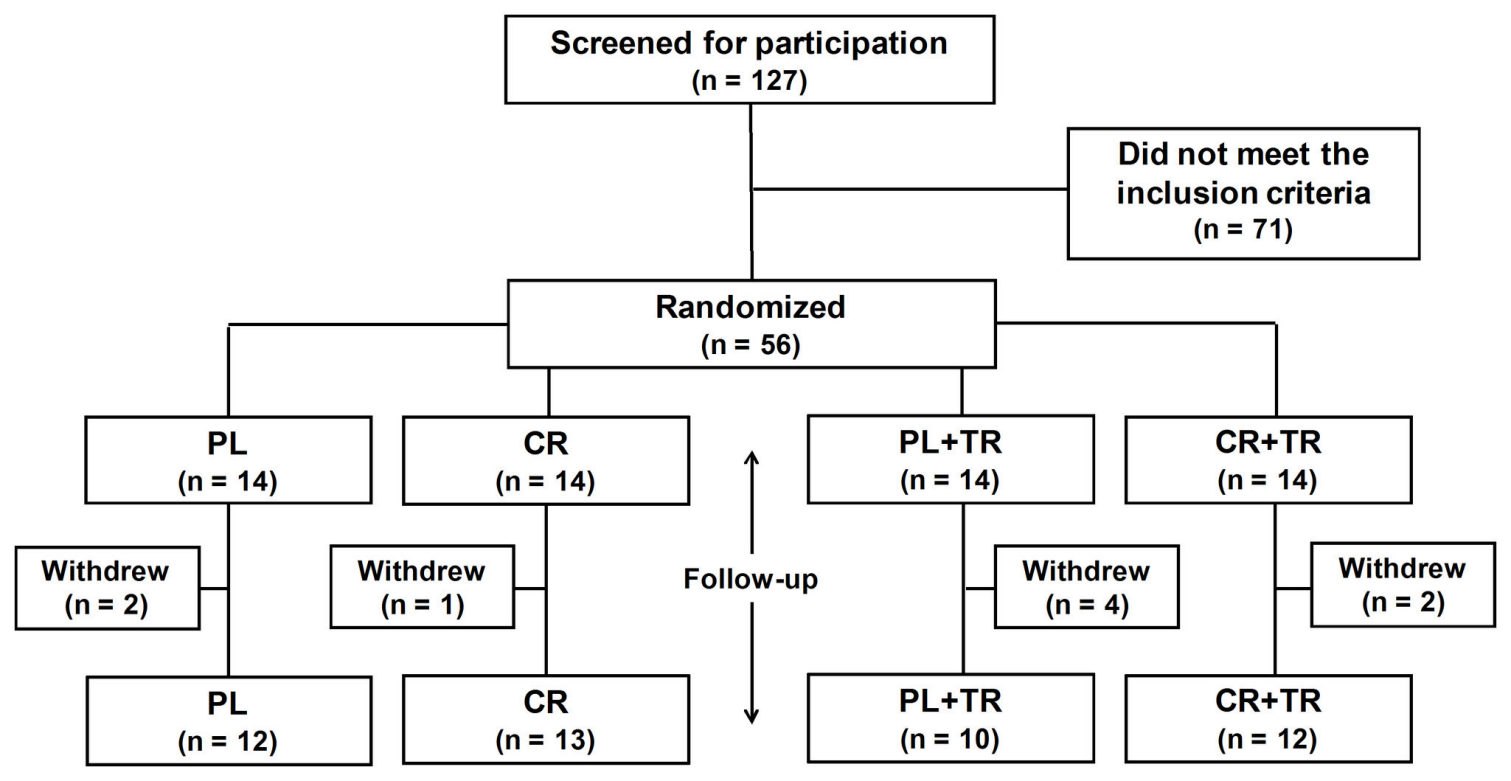
Fluxogram of subjects. PL = placebo supplementation, CR = creatine supplementation, PL + ST = placebo supplementation associated with strength training and PL+ST = creatine supplementation associated with strength training group.

Figure 2 illustrates the flow of participants. One hundred and twenty seven volunteers were screened for participation and 56 met the inclusion criteria and were included in this study. Nine participants withdrew for personal reasons (i.e., motivational or financial issues) during the follow-up and refused to perform the posttests (Placebo = 2; Creatine = 1; Placebo + Strength training = 4 and Creatine + Strength training = 2). Therefore, 47 subjects completed the follow-up and were analyzed (Placebo = 12; Creatine = 13; Placebo + Strength training = 10 and Creatine + Strength training = 12). Table 1 shows the main baseline characteristics of the participants.

**Table 1:** Main participants’ characteristics at baseline.

**Variable**	**Placebo (n = 12)**	**Creatine (n = 13)**	**Placebo + Strength training (n = 10)**	**Creatine + Strength training (n = 12)**	**p**
Age (years)	67.3 ± 5.6	66.9 ± 4.9	63.9 ± 3.8	66.4 ± 5.6	0.42
Height (m)	1.54 ± 0.09	1.53 ± 0.06	1.54 ± 0.05	1.56 ± 0.07	0.75
Weight (kg)	63.3 ± 8.9	65.4 ± 11.7	62.9 ± 5.9	68.4 ± 5.5	0.40
IMC (kg/m^2^)	27.0 ± 5.8	27.8 ± 4.8	26.5 ± 1.8	28.2 ± 1.7	0.75
Schooling (years)	7.3 ± 3.1	7.2 ± 4.0	6.3 ± 2.8	6.6 ± 3.5	0.90
Geriatric Depression scale (0 - 15)	1.9 ± 1.8	3.8 ± 2.3	3.7 ± 3.7	2.3 ± 1.9	0.16
MMSE (0 - 30)	26.3 ± 2.0	25.6 ± 2.4	27.3 ± 1.5	26.7 ± 3.0	0.36

No statistically significant differences between groups were observed at baseline.

### Assessment of blinding and adherence to supplementation and training

The number of the participants who correctly guessed the supplement was 8 (66.6%), 9 (69.2%), 6 (60.0%) and 8 (66.6%) for the PL, CR, PL+ST, and CR+ST groups, respectively. No significant differences between groups were noted (p = 0.77). The self-reported adherence to the supplementation protocol was 100%. The adherence to the strength training was 83.9 ± 6.1% and 84.4 ± 8.0% for the PL+ST and CR+ST groups, respectively. No significant differences between groups were observed (p = 0.86).

### Food intake and self-reported adverse events

No significant differences were observed for total energy, carbohydrate, lipid, and protein intake between groups (p > 0.05 for all variables; Table 2). There were no self-reported adverse events throughout the trial.

**Table 2:** Food intake at baseline and after 24 weeks of intervention.

**Variable**	**Placebo (n =12)**			**Creatine (n = 13)**			**Placebo + Strength Training (n = 10)**			**Creatine + Strength Training (n = 12)**			
***(time or score range)***	**PRE**	**POST-24**	**Δ (95% CI)**	**PRE**	**POST-24**	**Δ (95% CI)**	**PRE**	**POST-24**	**Δ (95% CI)**	**PRE**	**POST-24**	**Δ (95% CI)**	**p**
Total energy (kcal)	1411 ± 213	1518 ± 344	107 (-162 to 376)	1315 ± 324	1334 ± 358	19 (-285 to 323)	1438 ± 343	1343 ± 469	-95 (-506 to 316)	1436 ± 268	1444 ± 498	8 (-348 to 364)	0.86
Carbohydrates (g)	192 ± 41	211 ± 54	19 (-26 to 64)	162 ± 50	163 ± 43	1 (-41 to 430	189 ± 57	176 ± 57	-13 (-70 to 44)	189 ± 43	196 ± 93	7 (-58 to 71)	0.12
Carbohydrates (%)	54 ± 8	56 ± 10	2 (-7 to 11)	49 ± 6	49 ± 6	0 (-5 to 5)	52 ± 8	53 ± 7	1 (-7 to 9)	53 ± 9	52 ± 7	-1 (-8 to 6)	0.65
Protein (g)	59 ± 17	70 ± 18	11 (-6 to 28)	58 ± 16	63 ± 21	5 (-12 to 22)	58 ± 18	56 ± 13	-2 (-18 to 14)	56 ± 14	56 ± 20	0 (-15 to 15)	0.90
Protein (%)	16 ± 4	19 ± 5	3 (-1 to 7)	17 ± 2	19 ± 3	2 (-0 to 4)	16 ± 4	17 ± 4	1 (-3 to 5)	16 ± 3	16 ± 5	0 (-4 to 4)	0.49
Fat (g)	46 ± 13	44 ± 19	-2 (-17 to 13)	49 ± 12	47 ± 13	-2 (-13 to 9)	48 ± 14	51 ± 24	3 (-17 to 23)	55 ± 18	48 ± 13	-7 (-21 to 7)	0.52
Fat (%)	29 ± 7	25 ± 8	-4 (-11 to 4)	33 ± 6	32 ± 6	-1 (-6 to 4)	30 ± 8	34 ± 9	4 (-5 to 13)	34 ± 10	31 ± 7	-3 (-10 to 4)	0.30

No significant differences were observed between groups for any variable. Abbreviations and symbols: PRE = baseline; POST-24: after 24 weeks; **Δ** = absolute delta change; CI = confidence interval.

### Muscle strength

After the intervention, the CR+ST group had superior gains in 1-RM leg press (+18.7%) than the PL (-0.6%) and the CR groups (+1.6%) (p = 0.003 and p = 0.01, respectively), but not than the PL+ST group (+10.6%, p = 0.42). Both the trained groups (i.e., CR+ST: +10.0%, and PL+ST: +9.5%) showed greater strength in 1-RM chest-press than the PL group (-8.9%) (p = 0.002 and p = 0.02, respectively). However, there were no significant differences between the CR group (+0.3%) and others (p > 0.05).

### Emotional and cognitive measures

**Figure 3:**
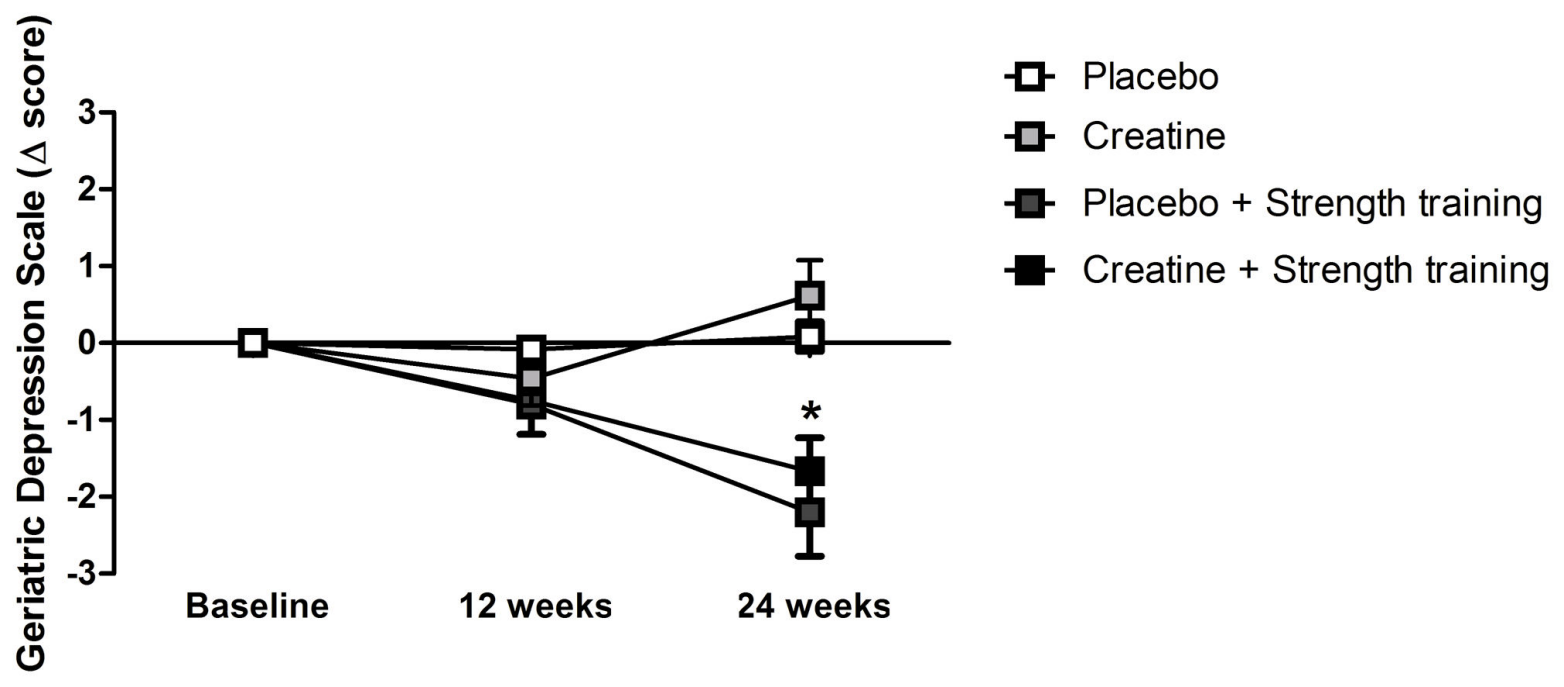
Delta changes in Geriatric Depression Scale at baseline and after 12 and 24 weeks of intervention. * denotes statistically significant differences at 24 week in the comparisons between the trained *versus* non-trained groups (i.e., Placebo + Strength training and Creatine + Strength training *versus* Placebo and Creatine; p < 0.05).

Figure 3 shows the delta changes for the Geriatric Depression Scale. No differences were detected after 12 weeks of intervention (p = 0.10). However, after 24 weeks of intervention, the PL+ST (absolute delta change = -2.2; 95% CI = -5.2 to 0.8) and CR+ST (absolute delta change = -1.7; 95% CI = -3.0 to -0.4) groups had significant reductions in depression scores when compared with either the PL group (absolute delta change = +0.1; 95% CI = -1.4 to 1.6; p = 0.01 and p = 0.0009, respectively) or the CR (absolute delta change = +0.6; 95% CI = -1.6 to 2.8; p = 0.0002 and p < 0.0001, respectively) groups. No significant differences were observed between the PL and CR groups (p = 0.60) nor between the PL+ST and CR+ST groups (p = 0.84).

The data regarding cognitive performance are presented in Table 3. No significant differences were observed for any of the variables throughout the intervention (data at 12 week are not shown).

**Table 3:** Cognitive performance at baseline and after 24 weeks of intervention.

**Variable**	**Placebo (n =12)**			**Creatine (n = 13)**			**Placebo + Strength Training (n = 10)**			**Creatine + Strength Training (n = 12)**			
***(time or score range)***	**PRE**	**POST-24**	**Δ (95% CI)**	**PRE**	**POST-24**	**Δ (95% CI)**	**PRE**	**POST-24**	**Δ (95% CI)**	**PRE**	**POST-24**	**Δ (95% CI)**	**p**
**MMSE** (0 to 30)	26.1 ± 1.9	26.8 ± 2.3	0.7 (-1.1 to 2.5)	25.6 ± 2.4	26.6 ± 1.9	1 (-0.7 to 2.7)	27.1 ± 1.7	26.9 ± 2.4	-0.2 (-2.1 to 1.7)	27.5 ± 1.8	27.8 ± 2.6	0.3 (-1.6 to 2.2)	0.12
**BBCS**													
Naming (0 to 10)	10.0 ± 0.0	10.0 ± 0.0	-	10.0 ± 0.0	10.0 ± 0.0	-	10.0 ± 0.0	10.0 ± 0.0	-	10.0 ± 0.0	10.0 ± 0.0	-	-
Incidental memory (0 to 10)	6.3 ± 1.7	7.1 ± 1.8	0.8 (-0.7 to 2.3)	6.6 ± 1.3	8.2 ± 1.5	1.6 (0.5 to 2.7)	6.3 ± 1.3	7.8 ± 0.8	1.5 (0.5 to 2.5)	5.9 ± 1.6	7.8 ± 1.2	1.9 (0.7 to 3.1)	0.47
Immediate memory (0 to 10)	8.8 ± 1.1	8.8 ± 1.2	0.0 (-1.0 to 1.0)	8.8 ± 1.1	9.4 ± 0.7	0.6 (-0.2 to 1.4)	8.7 ± 0.8	9.2 ± 1.0	0.5 (-0.4 to 1.4)	8.6 ± 1.2	9.5 ± 0.7	0.9 (0.1 to 1.7)	0.88
Learning (0 to 10)	9.3 ± 1.1	9.9 ± 0.3	0.6 (0.1 to 1.3)	9.5 ± 0.9	9.8 ± 0.4	0.3 (0.3 to 0.9)	9.4 ± 0.5	9.8 ± 0.4	0.4 (0.0 to 0.8)	9.7 ± 0.7	10.0 ± 0.0	0.3 (-0.1 to 0.7)	0.09
Delay recall (0 to 10)	9.3 ± 1.0	8.9 ± 0.9	-0.4 (-1.2 to 0.4)	8.8 ± 1.1	9.4 ± 0.8	0.6 (-0.2 to 1.4)	8.8 ± 1.4	9.4 ± 0.7	0.6 (-0.4 to 1.6)	8.8 ± 1.0	9.7 ± 0.5	0.9 (0.2 to 1.6)	0.07
**Digit span test**													
Forward order (0 to 7)	5.3 ± 1.1	5.0 ± 1.0	-0.3 (-1.2 to 0.6)	5.3 ± 0.9	5.6 ± 1.1	0.3 (-0.5 to 1.1)	4.9 ± 0.9	4.7 ± 1.1	-0.2 (-1.1 to 0.7)	5.0 ± 1.0	5.7 ± 1.0	0.7 (-0.2 to 1.6)	0.99
Backward order (0 to 7)	3.1 ± 0.8	3.3 ± 0.8	0.2 (-0.5 to 0.9)	3.4 ± 1.0	3.7 ± 0.9	0.3 (-0.5 to 1.1)	3.2 ± 0.8	3.2 ± 0.7	0.0 (-0.7 to 0.7)	3.6 ± 1.2	3.8 ± 1.1	0.2 (-0.8 to 0.2)	0.90
**Stroop conditions**													
Color (s)	16.6 ± 4.5	14.7 ± 2.9	-1.9 (-5.1 to 1.3)	15.5 ± 4.1	16.2 ± 3.7	0.7 (-2.5 to 3.9)	17.3 ± 3.9	15.7 ± 3.2	-1.6 (-5.0 to 1.8)	15.5 ± 5.0	14.3 ± 4.0	-1.2 (-5.0 to 2.6)	0.68
Non-color word (s)	24.7 ± 7.9	23.3 ± 5.6	-1.4 (-7.2 to 4.4)	23.5 ± 9.4	22.2 ± 6.0	-1.3 (-7.7 to 5.1)	26.0 ± 7.5	21.4 ± 3.5	-4.6 (-10.1 to 0.9)	20.7 ± 5.1	19.5 ± 5.8	-1.2 (-5.8 to 3.4)	0.16
Color word (s)	37.4 ± 9.4	31.6 ± 8.8	-5.8 (-13.5 to 1.9)	32.7 ± 10.8	32.8 ± 11.1	0.1 (-8.8 to 9.0)	36.8 ± 7.3	30.1 ± 6.0	-6.7 (-13.0 to -0.4)	33.9 ± 13.1	33.7 ± 13.7	-0.2 (-11.6 to 11.2)	0.88
**Trail Making test**													
Part A (s)	52 ± 21	60 ± 29	8 (-13 to 29)	53 ± 28	49 ± 20	-4 (-24 to 16)	64 ± 21	52 ± 19	-12 (-31 to 7)	40 ± 14	38 ± 9	-2 (-12 to 8)	0.52

No significant differences were observed between groups for any variable. Abbreviations and symbols: PRE = baseline; POST-24: after 24 weeks; **Δ** = absolute delta change; CI = confidence interval; BBCS = brief battery of cognitive screening.

We have also performed a sensitivity analysis by excluding those subjects with Geriatric Depression Scale score of 0 (n = 3). The between-group differences remained exactly the same. The PL+ST and CR+ST groups had greater reductions in the Geriatric Depression Scale scores when compared with either the PL group (p = 0.0013 and p = 0.013, respectively) or the CR group (p < 0.0001 and p = 0.0002, respectively) groups. No differences were found between the trained groups (i.e., PL+ST *vs*. CR+ST, p = 0.895).

### Adverse events

There were no self-reported side effects throughout the study. Clinical evaluations did not reveal adverse events related to creatine supplementation or strength training.

## Discussion

To our knowledge, this is the first randomized controlled trial to investigate the combined effects of creatine supplementation and strength training on emotional and cognitive measures in elderly individuals. Our main findings are as follows: i) creatine supplementation *per se* or additively to strength training does not promote any benefit on selected aspects of cognitive function and emotional state; and ii) strength training *per se* is able to improve emotional measures, but not cognitive function.

A high turnover of ATP is necessary to match the fluctuating energetic demand in the brain. In this respect, the phosphorylcreatine system has been thought to have a pivotal role to normal cerebral metabolism [41]. In fact, changes in brain energy metabolism, neuronal plasticity, and cellular resiliency are associated with the pathogenesis of depressive disorders [20,22,42–45]. Moreover, evidence indicates that brain bioenergetics and cell survival pathways are potential therapeutic targets for long-term clinical relief and symptom remission [46–49]. In this context, creatine supplementation has emerged as a potential dietary intervention capable of buffering metabolic processes, thereby preventing energy exhaustion and neuronal death. Interestingly, there is a large body of literature showing that alterations in brain creatine and phosphorylcreatine levels are associated with depression [17–22]. Furthermore, preliminary findings suggested that creatine supplementation improves depressive symptoms in humans [15,16]. For instance, patients with treatment-resistant depression [15] or post-traumatic stress disorders associated or not with comorbid depression [16] supplemented with creatine monohydrate for 4 weeks reported elevated mood on the Hamilton Depression Rating Scale. Conversely, in the current study, creatine supplementation, combined or not with strength training, failed to improve emotional measures in elderly individuals. The possible explanation for these conflicting findings may rely in differences among studied populations. Whilst creatine supplementation has been shown to be effective in ameliorating symptoms in patients with diagnosed refractory depression [15] or other severe psychiatric disorders [16], it is possible that this dietary supplement has limited (if any) effect in apparently mental healthy elderly individuals.

Importantly, we performed a *post-hoc* sensitivity analysis excluding the subjects with the Geriatric Depression Scale score of 0 and did not find any significant effect of creatine supplementation, which partially suggests that a "ceiling effect" was not responsible for the absence of beneficial effect of this dietary supplement in this study. However, as only 6 subjects had Geriatric Depression Scale scores higher than 6 (which is the cut-off point to suggest depression), we were unable to evaluate the effects of creatine in "more depressed" participants, thus warranting further studies with depressive older subjects.

Further to the speculation that creatine supplementation could exert any effect on emotional state, we also hypothesized that this dietary supplement could promote beneficial effects upon cognitive function. In fact, previous studies showed that supplementation can improve selected aspects of cognitive performance in young individuals [13] as well as in elderly people [50]. Furthermore, creatine intake was also shown to alleviate mental fatigue induced by stressor stimulus, such as mathematical calculus [13] and sleep deprivation [14]. Additionally, a recent study demonstrated that in vegetarians, but not in omnivorous, creatine supplementation improved memory. Moreover, both vegetarians and meat eaters experienced decreased variability in the responses to a choice reaction-time task [51]. However, in the current study, creatine supplementation was unable to promote any significant improvement in several aspects of cognitive function. Although it is difficult to reconcile these conflicting findings, a few explanations do exist. Overall, the most important effects of creatine supplementation on cognitive function have been seen either under i) stressing conditions, such as sleep deprivation, exhausting exercise and mental fatigue [13,14] or ii) vegetarian diet, which might lead to partial depletion in brain creatine content [52]. Therefore, it is possible to speculate that creatine supplementation does not benefit cognitive function in healthy individuals not subjected to stressing conditions. Additional studies with elderly subjects with mild or severe cognitive impairment should be performed. Moreover, an overview of the literature [33] points to other factors that may be also responsible for the divergent results with respect to the creatine effects on cognition, including the variation in the follow-up period of supplementation (i.e., 7 d to 24 weeks), the age of the participants (i.e., adults *versus* elderly), the different types of cognitive tests applied (i.e., memory, time-to-reaction, intelligence, attention, verbal fluency tests), and the experimental design (i.e., nonrandomized *versus* randomized controlled trials).

Independently of creatine supplementation, the strength training program led to improvements in emotional measures. These data partially supports previous findings showing improvements in mood profile in old individuals after 24 weeks of strength training [4,31]. Interestingly, however, we did not find any effect of strength training upon cognition, contrasting previous observations of better cognitive performance (e.g., memory, recognition and attention) following strength exercises [30–32]. Cassilhas et al. (2007) suggested that the main mechanism underling the improvements in cognition seen in their study likely involves an exercise-mediated increased secretion of Insulin Growth Factor-1 (IGF-1), a hormone that modulates the brain-derived neurotrophic factor (BNDF), which, in turn, is believed to exert neuroprotective actions. Since mechanist analyses were beyond the scope of the current study, further studies should examine whether strength training affects cognitive function in old individuals as well as the exact mechanism underlying this response.

Finally, creatine has been considered one of the few dietary supplements capable of increasing muscle strength in elderly individuals. Furthermore, there is some evidence suggesting that strength training combined with creatine supplementation promotes greater improvements in muscle function than strength training alone [33]. Our present findings do not fully support these assumptions, since the association of creatine supplementation and training did not elicit superior strength gains than training alone. One may speculate that creatine supplementation failed to increase muscle creatine content to a level that would allow the observation of improvements in muscle function. In fact, this argument appears to be also applicable to the case of the central nervous system; if creatine supplementation did not promote sufficient increase in brain creatine content, one might expect only minor (if any) improvements in cognitive and emotional parameters. Therefore, we recognized that the lack of tissue (i.e., brain and muscle) creatine content assessment following creatine supplementation is the major limitation of this study. In fact, as stressed by Rawson et al. 2011, no study has been done to ensure that creatine supplementation is able to penetrate the blood-brain-barrier and, consequently, increase brain creatine content in old individuals. Further studies approaching this issue are of great relevance.

To conclude, creatine supplementation *per se* or additively to strength training does not promote any benefit on cognitive function and emotional measures in apparently healthy elderly individuals. In addition, strength training *per se* was shown to improve emotional state and muscle strength, but not cognition, in our sample. Further studies involving frailer older individuals undergoing strength training and ingesting creatine supplementation are necessary to test the efficacy of this intervention.

## Supporting Information

Checklist S1 **CONSORT Checklist.**
(DOC)

Protocol S1 **Trial Protocol.**
(DOC)

Protocol S2 **Trial Protocol (Portuguese).**
(DOC)
