# Supplementary material for: Creatine Supplementation Associated or Not with Strength Training upon Emotional and Cognitive Measures in Older Women: A Randomized Double-Blind Study
Source: PLoS One. 2013 Oct 3;8(10):e76301. doi: 10.1371/journal.pone.0076301 (PMC3789718; doi:10.1371/journal.pone.0076301)
Supplement: Protocol S1 — Trial Protocol. (DOC) [file pone.0076301.s002.doc]

**University of São Paulo**

**Laboratory of Bone Metabolism, Rheumatology Division, School of Medicine**

**Laboratory of Evaluation and Conditioning in Rheumatology, Rheumatology Division, School of Medicine**

**Laboratory of Applied Nutrition and Metabolism, School of Physical Education and Sports**

**EFFECTS OF STRENGTH TRAINING ASSOCIATED OR NOT WITH CREATINE SUPPLEMENTATION IN COGNITIVE FUNCTION OF ELDERLY**

**Principal Investigator**

Prof. Rosa Maria Rodrigues Pereira, PhD

**Investigators:**

Christiano Robles Rodrigues Alves

Prof. Bruno Gualano, PhD

São Paulo

2010

EFFECTS OF STRENGTH TRAINING ASSOCIATED OR NOT WITH CREATINE SUPPLEMENTATION IN COGNITIVE FUNCTION OF ELDERLY

The literature provides evidence highlighting the protective role of exercise on cognitive function decline. Main perspective relate to the aerobic exercise. However, some researchers also indicate the beneficial influence of strength exercise in cognition and suggest possible mechanisms for it to occur. Another non-pharmacological strategy proposal aimed at attenuate cognitive losses is the creatine supplementation. This amine seems to have an important therapeutic role and some authors indicate its influence on the central nervous system. However, no study to date has investigated the effect of strength exercise training associated with creatine supplementation on cognitive function in the elderly. Thus, the aim of this study is to evaluate a possible synergy effect between both strategies. We will carry out a double-blind, randomized, controlled trial. The subjects will be divided into four groups: Placebo + Training (RT + P), Training + Creatine (Cr + TR), Placebo (P) and creatine (Cr). For 24 weeks the subject groups TR and TR + P + Cr will undergo physical training, while groups P and Cr are instructed not to join in any physical activity program. Before, after 12 and after 24 weeks of intervention, all groups will perform tests of muscle strength, nutritional assessment and cognitive testing.

**1. Introduction and justification**

The aging process can be explained by the addition of organic amendments, functional and psychological in humans. These changes are inevitable and also can be aggravated by numerous diseases later in life (Papaléo, 2002). Memory, language, ability association, comparison, synthesis and attention they deserve special attention because they are directly linked to the quality of life of the elderly population. In the aging process occurs a decrease in the ability to recall new information, the handling thereof, the ability to perform multiple tasks simultaneously, finally, executive functions are affected in general during aging compared to young adults (Spirduso et al, 2005).

Given the increasing elderly people population, few researchers have investigated possible strategies to mitigate the losses of cognitive functions. In this context, highlights nonpharmacological strategies such as aerobic physical training (Blackwood et al, 1998; Kashihara et al, 2009; Davranche et al, 2009) and strength (Liu-Ambrose et al, 2009) and the creatine supplementation (McMorris et al, 2007; Andres et al, 2008; Bender, 2006).

The studies aimed to investigate the effects of aerobic exercise on cognitive function are not recent. Since 1980, papers showed influence of aerobic exercise upon cognition. Although there is still a great discussion in this area, especially about the intensity of these exercises should be performed (Kashihara et al, 2009), some studies with moderate aerobic exercise (60% of VOmax) demonstrate, by means of infrared spectroscopy, which is the positive effect on cognition, as it improves regional cerebral blood flow and consequently this leads to a better supply of oxygen and glucose in the brain area that controls cognitive functions (Comp et al, 1996). Moreover, acute aerobic exercise can alter the levels of neurotransmitters in the central nervous system, such as acetylcholine, dopamine, noradrenaline, adrenaline, adrenocorticotropic hormone (ACTH) and vasopressin, can activate again cognitive function (Spirduso, 1980 ; Radosevich et al, 1989; Rikli et al, 1991). Further details can be found in a recent article of Kashihara et al. (2009).

Strength training provides various metabolic and hemodynamic adaptations in the body, such as the reduction of abdominal fat, improvements in plasma concentrations of triglycerides, increased high density lipoprotein-cholesterol (HDL-C) and glycemic control (APM Guttierres Martins & JCB , 2008). Other important adjustments are changes in the rate of elimination of glucose, increasing power to muscle glycogen content, GLUT4 receptor in muscle, insulin sensitivity and doing a normalization of glucose tolerance. It is still important to note that the hemodynamic benefits of strength training leads to the tendency to reduce systolic and diastolic blood pressure by promoting the secretion of vasodilating substances and participation as a valuable component for angiogenesis, or formation of new capillaries. Umpierre & Stein commented the apparent effectiveness of strength training on the maintenance of peripheral blood flow in elderly, which can help to minimize the functional limitations present in aging or in different pathological conditions. In addition, strength training increases muscle mass and maximal muscle strength and can also alleviate the advancement of sarcopenia (Umpierre & Stein, 2007).

Recently, it has been shown that strength training also has an important role on cognitive function. Cassilhas et at (2007) demonstrated, through batteries of tests Wechsler Adult Intelligence Scale III (WAIS III), Wechsler Memory Scale-Revised (WSM-R), Toulouse-Pieron's concentration attention test that 24 weeks of strength training in two different intensities, 50% and 80% of 1RM in two sets of eight reps each, can improve cognitive performance in older men. Furthermore, the researchers found an increase in serum concentrations of Insulin-like Growth Factor (IGF-I) in groups trained in strength. It is known that the increase in IGF-I decreases the plasma concentration of homocysteine, an amino acid which is derived from sulphurous demethylation of methionine which at high levels can cause brain damage and neuropsychiatric disorders (Liu-Ambrose et at, 2009; Ducloux et at , 2002). Homocysteine ​​is pro-atherogenic and pro-thrombotic, increasing the risk of stroke and may have a direct neurotoxic effect (P Sachdev, 2004). IGF-I also modulates the levels of acetylcholine in the hippocampus that play important roles in the development of Central Nervous System (Arwert et at, 2005). In another study, Perrig-Chiello et al showed that 8 weeks of strength training significantly improved self-care, self-centered thoughts, memories, memory and anxiety in elderly individuals. In a recent review (2009), Liu-Ambrose et at commenting on the need for more research to clearly define the role of resistance exercise in preventing cognitive decline that affects the elderly.

Another strategy supposedly able to slow the loss of cognition is creatine supplementation. This amine synthesized in the liver, kidney and pancreas, or obtained via the feed, is primarily (95%) in skeletal muscle, the remainder being distributed in the intestine, smooth muscle, testis and brain (Wyss et at, 2000). Creatine is found in the human body in free forms (60 to 70%) and phosphorylated (30-40%). One of the major roles of CK-PCr system is energy transfer from mitochondria to the cytosol, especially in tissues where energy demand is high, such as muscle and brain (Gualano et al, 2009).

The brain accounts for approximately 20% of total energy consumption of the body (Shulman et at, 2004). Recently, it has been found that creatine orally administered is able to overcome the blood brain barrier and increase brain concentrations of this substrate (Andres et al, 2008). In a recent literature review, Andres et al point out that the system fosforilcreatina (PCR) plays a key role in energy metabolism of the brain due to resynthesis of adenosine triphosphate (ATP) by the creatine kinase (CK). Memory consolidation, for example, is performed in the hippocampus and requires a lot of energy-dependent ATP hydrolysis (McMorris et al, 2007). Furthermore, the results of some studies indicate that creatine supplementation can improve cognitive function in healthy young (Watanabe et al. 2002) and aged (McMorris et al. 2007). Besides the important function of power supply to the brain proposed to explain the best performance in math young adults (24.3 + / - 9.1 years), Watanabe et al demonstrated by means of infrared spectroscopy, that supplementation with Creatine, as well as physical exercise (Kashihara et al, 2009) appears to also be able to increase the cerebral oxygenation (Watanabe et al, 2002). However, the mechanisms by which this increase oxygenation act on cognition were still little explored.

It is known that the elderly require more energy to perform cognitive tasks compared to younger subjects (Behzadi & Liu, 2005; Toescu, 2005). Although studies using magnetic resonance spectroscopy show that the level of brain creatine tends to increase with age, this increase is not enough to compensate for cognitive losses, raising the possibility of creatine supplementation attenuate such a framework (McMorris T et al, 2007). In addition, Ellis and Rosenfeld (2004) demonstrated that creatine is capable of increasing the antioxidant and McMorris et al, 2007 comment about an increase in brain concentration of dopamine, a neurotransmitter that is essential in cognitive tasks, as previously discussed. In vitro models have been shown beneficial effects of creatine on the reuptake of glutamate, which is known to be neurotoxic in high concentrations (RH Andres et al, 2008). Considering these data together, we can say that creatine supplementation plays an important role in cognitive function and promising.

Given the benefits promoted by strength training and creatine supplementation on cognition, the purpose of this study is to investigate the combined effects of both strategies on the cognitive function in elderly.

**2. Purpose**

**2.1. General purpose**

To investigate the effects of strength training associated or not with creatine supplementation on cognitive function in elderly.

**2.2. Specific purpose**

To measure cognition and strength performance before and after 12 and 24 weeks in elderly.

**3. Methods**

**3.1. Experimental design**

A clinical, randomized, double-blind, placebo-controlled study will be conducted. In order to check the eligibility criteria, all subjects will undergo medical evaluation. 60 volunteers will be randomly divided into 4 groups: Placebo + Training (RT + P), Training + Creatine (Cr + TR), Placebo (P) and creatine (Cr).

Over 24 weeks, the subjects of the groups TR and TR + P + Cr will undergo strength training (see item 3.6), while those of groups P and Cr are instructed not to enter any physical activity program. In the pre-intervention period (PRE), after 12 weeks (POST 12) and after 24 weeks (POST 24) volunteers conduct cognitive tests, strength tests and blood samples to measure the desired variables.

**3.2. Samples**

60 volunteers will be selected to go through interviews and medical evaluation (including exercise testing) analysis framework for the inclusion criteria of the study, all of whom have signed a term of informed consent, according to the norms of the Ethics Committee.

*Inclusion Criteria:*

• Seniors: 60 to 80 years

• Females

• There is no practitioner of physical training at least one year.

• Not supplemented with creatine for at least 6 months.

*Exclusion Criteria:*

• Individuals who do not attend school and unable to read a simple sentence.

• Bouts in locomotor or cardiovascular diseases that are limiting to practice the exercises

• Glomerular filtration rate less than 30ml/kg/min

• Smokers

**3.3. Ergometric Test**

Volunteers will receive, in writing, all relevant instructions to the execution of the test. During the test, the pressure responses will be verified through mercury sphygmomanometer; chronotropic and ECG, electrocardiogram through; perceived exertion, with the aid of the Borg scale. All tests will be conducted under the supervision of a cardiologist.

**3.4. Nutritional Assessment**

Participants will be instructed not to change their food consumption during the study. To assess possible changes are nutritional assessments in periods PRE and POST 12. The evaluation will consist of analysis of food diary for three days, two weekdays and one weekend day (Scagliusi F, 2003).

**3.5. Supplementation**

Supplementation will be held according to double-blind, meaning neither the researchers nor the volunteers will know if they are ready ingesting creatine or placebo. A nutritionist will be responsible for the randomization of subjects in both groups and designation according to your own choice: Group 1 and Group 2. The groups will be revealed only after statistical analysis.

During the first five days, volunteers will receive a supplement (20g per day) divided into four plastic bags of the type "zip lock" bag within a properly identified with your name. After six days each patient will consume the contents of one bag per day (5 g). All volunteers will be instructed to ingest supplementation accompanied by juice, preferably during a meal. Supplementation should not be consumed with caffeine.

Will be provided to each patient a monthly table which shall contain the time the supplement was ingested, monitoring (orange juice, for example) and a corresponding label to the bag "zip-lok". Each bag is labeled with a tag called: Group 1 or Group 2.

This method is an adaptation of a previous study (Gualano et al. 2008), where reports indicate a 100% adherence to the protocol.

**3.6. Muscle strength and function assessment**

Muscle strength will be determined by testing one repetition maximum (1RM). The test will be conducted in two different outfits, which are: chest press and leg press. There will be a specific heating of 8 reps with a load of approximately 50% and after 2 minutes rest, 3 repetitions with approximately 70% of 1RM estimated for the individual. After 3 minutes of specific warm-up, the individual must make 5 attempts a repeat with 3 minutes rest between them. To avoid the influence of motor learning and neural adaptations of the movement will be performed three familiarization sessions previously reported in the literature as sufficient to occur a stabilization measure (Phillips et al, 2004).

To complement the assessment of muscle strength tests will be performed isometric contraction (handgrip and lumbar traction) with dynamometers. These tests should be done two times and on different days (48 hours off) where, every day, must be met 3 attempts for each dynamometer. The handgrip strength is measured with the dominant arm of the individual lying beside the body and lumbar traction the individual should position yourself standing on the platform of the dynamometer with the knee extended and flexed trunk forward at an angle of approximately 120 º from this position the individual must apply force to try to return the position of "standing" (Brown, 2003).

The functional tests are designed to assess the strength gain to daily activities. Two different tests will be applied twice each day and again on different. The first test is an adaptation of functional test "Timed-stands test" (Newcomer et al, 1993), where, in our case, it will consist of the number of times that the individual can stand up and sit in a chair again forming a angle of approximately 90 degrees at the knee for 30 seconds. The second test is called "Timed up-and-go" (Podsiadlo et al, 1991) where the patient should get up from a chair, walk three meters forward and return to the chair. The total time spent will be measured.

**3.7. Strength Training**

Strength training consists of two sessions per week during the 24 week study. A complete training session (warming, and stretching exercises force) will last about 50 minutes and it is important to emphasize that to evaluate the adhesion of subjects will be monitored closely the presence thereof.

In each session there will be a general warm-up treadmill, about 5 minutes, followed by a specific warm-up (50% of 1RM) before each exercise. After heating, should be performed 3 sets of 8 to 12 repetitions maximum for eight different exercises, always respecting a rest of 90 seconds between sets.

The seven exercises were filed in order to work the large muscle groups. Initially a training session will be comprised of the following exercises: bench press, Leg Press, Row, Leg extension, Pulley, Free Squat and Abdominal crunch. After the exercises will be held stretches of mild to moderate intensity for the major muscle groups.

**3.8. Cognitive assessment**

The cognitive assessment will be measured by cognitive tests: MMSE, Trails Test (forms A and B), Stroop Test, Word List of Gerad and the Geriatric Depression Scale Short.

  The MMSE is composed of items that assess orientation, timeline, immediate memory, recall memory, attention and language, with a total score of 30 points (Folstein et al., 1975; Brucki et al., 2003). Furthermore, we will use the Test Tracks A and B. Track Test A consists in connecting, in ascending order, and by a continuous line, all numbers (1,2,3 ...) forming a figure. The Test Track B is requested to examine that alternately connect numbers and letters (1-A, 2-B, 3-C ...). Both aim to assess attention, sequencing, mental flexibility, visual search and motor function, and the Test Track B still requires greater attention span and ability to make conceptual changes alternating (Mota et al, 2008). To assess selective attention, processing speed, task switching, ability to inhibit irrelevant stimuli and inhibition of responses will be used Stroop Test (Spreen & Strauss, 1998). To assess the memory is used to CERAD Word List (Bertolucci et al., 2001). Still be applied to the Geriatric Depression Scale Short (Sheikh et al, 1986) to assess depression.

**3.9. Body composition**

The assessment of body composition (lean mass, fat mass and bone mineral content) will be made by DXA using the Hologic Discovery appliance, pre and after 24 weeks of intervention.

**3.10. Place to conduct the experiments**

Physical training, nutritional assessment, exercise testing and the application of cognitive tests will be conducted in the Laboratory Assessment and Conditioning in Rheumatology at the Hospital. The assessment of body composition will be performed at the Laboratory of Bone Metabolism - Rheumatology (LIM-17) FMUSP.

**3.11. Statistical Analyses**

Data are expressed as mean ± standard deviation or percentage. The difference (final - initial) among the four groups will be analyzed by ANOVA followed by Tukey test or Kruskal-Wallis test (nonparametric). The chi-square test or Fisher's exact test is used to compare categorical variables. The level of significance to reject the null hypothesis will be p ≤ 0.05.

**4. Execution Schedule**

After approval by the ethics committee of the total project duration will be 24 weeks. According to this distribution:

• Volunteer Recruitment and Selection - 8 weeks

• Collects and tests PRE - 8 weeks

• Intervention - 12 weeks

• Collects and tests POST 12 weeks

• Intervention - 12 weeks

• Collects and tests POST 24 weeks

• Analysis and publication of data - 8 weeks

**5. Deviations from the original protocol**

The following modifications were implemented due to either the technical issues or the need of methodological adjustments:

1) Inclusion of additional cognitive questionnaires, including a specific one to assess depression.

2) Exclusion of DXA and muscle function measurements.

All the changes were in full accordance with the ethical procedures established by the National Brazilian Ethics Committee (CONEP).

**6. References**

Andres RH, Ducray AD, Schlattner U, Wallimann T, Widmer HR. Functions and effects of creatine in the central nervous system. Brain Research Bulletin (2008) 76: 329–34

Arwert LI, Deijen JB, Drent ML. The relation between insulin-like growth factor I levels and cognition in healthy elderly: A meta-analysis. Growth Hormone & IGF Research (2005) 15:416–422

Atalaia-Silva KC, Lourenço RA. Tradução, adaptação e validação de construto do Teste do Relógio aplicado entre idosos no Brasil. Rev Saúde Pública (2008) 42(5): 930-7

Bender A, Koch W, Elstner M, Schombacher Y, Bender J, Moeschl M, Gekeler F, Muller-Myhsok B, Gasser T, Tatsch K, Klopstock T. Creatine supplementation in Parkinson disease: a placebo-controlled randomized pilot trial. Neurology (2006) 67: 1262–1264

Berg KO, Wood-Dauphinee SL, Williams JI, Gayton D. Measuring balance in the elderly: Preliminary development of an instrument. Physiotherapy Canada (1989) 41: 304-11

Blackwood SK, MacHale SM, Power MJ, Goodwin GM, Lawrie SM. Effects of exercise on cognitive and motor function in chronic fatigue syndrome and depression. J Neurol Neurosurg Psychiatry (1998) 65: 541–546

Brucki SMD, Nitrini R, Caramelli P, Bertolucci PHF, Okamoto IH. Sugestões para o uso do Mini-exame de Estado Mental no Brasil. Arq Neuropsiquiatr 2003;61(3-B):777-781

Bustamante SEZ , Bottino CMC, Lopes MA, Azevedo D, Hototian SR , Litvoc J, Jacob Filho W. Instrumentos combinados na avaliação de demência em idosos. Arq Neuropsiquiatr (2003) 61: 601-606

Brown LE & WEIR JP. ASEP Procedures Recommendation I: Accurate Assessment Of Muscular Strength And Power. JEPonline. 2001;4(3):1-21.

Cassilhas RC, Viana VA, Grassmann V, [Santos RT](http://www.ncbi.nlm.nih.gov/pubmed?term="Santos RT"%5BAuthor%5D&itool=EntrezSystem2.PEntrez.Pubmed.Pubmed_ResultsPanel.Pubmed_RVAbstract), [Santos RF](http://www.ncbi.nlm.nih.gov/pubmed?term="Santos RF"%5BAuthor%5D&itool=EntrezSystem2.PEntrez.Pubmed.Pubmed_ResultsPanel.Pubmed_RVAbstract), [Tufik S](http://www.ncbi.nlm.nih.gov/pubmed?term="Tufik S"%5BAuthor%5D&itool=EntrezSystem2.PEntrez.Pubmed.Pubmed_ResultsPanel.Pubmed_RVAbstract), [Mello MT](http://www.ncbi.nlm.nih.gov/pubmed?term="Mello MT"%5BAuthor%5D&itool=EntrezSystem2.PEntrez.Pubmed.Pubmed_ResultsPanel.Pubmed_RVAbstract). The impact of resistance exercise on the cognitive function of the elderly. Med Sci Sports Exerc (2007) 39:1401–7

Davranche K, McMorris T. Specific effects of acute moderate exercise on cognitive control. Brain and Cognition (2009) 69: 565–570

Ducloux D, Motte G, Nguyen NU, Abdelfatah A, Gibey R, Chalopin JM. Homocysteine, nutritional status and insulin in renal transplant recipients. Nephrol Dial Transplant (2002) 17: 1674–1677

Fava DC, Kristensen CH, Melo WV, Araújo LB. Construção e validação de tarefa de Stroop Emocional para avaliação de viés de atenção em mulheres com Transtorno de Ansiedade Generalizada. Paidéia (2009) 19 (43): 159-165

Fernandes RCL, Silva KS, Bonan C, Zaha SEV, Marinheiro LPF. Avaliação da cognição de mulheres no climatério com o Mini-Exame do Estado Mental e o Teste de Memória da Lista de Palavras. Cad. Saúde Pública (2009), Rio de Janeiro, 25 (9): 1883-1893

Folstein MF, Folstein SE, McHugh PR. Mini-Mental State: a practical method for grading the cognitive state of patients for clinician. J Psychiatr Res 1975;12:189-198.

Gomes CMA, Borges O. Qualidades Psicométricas de um conjunto de 45 testes. Fractal Revista de Psicologia (2008) 20: 195-208

Gualano B, Artioli GG, Poortmans JR, Lancha AH Jr. Exploring the therapeutic role of creatine supplementation. Amino Acids (2009)

Gualano B, Novaes RB, Artioli GG, Freire TO, Coelho DF, Scagliusi FB, Rogeri PS, Roschel H, Ugrinowitsch C, Lancha AH Jr. Effects of creatine supplementation on glucose tolerance and insulin sensitivity in sedentary healthy males undergoing aerobic training. Amino Acids (2008) 34: 245–250

Guttierres APM & Martins JCB. Effects of Resistance Training Over Metabolic Sydrome Risk Factors. Rev Bras Epidemiol. 2008; 11(1): 147-58

Kalache A, Veras RP, Ramos LR. O envelhecimento da população mundial: um desafio novo. Revista Saúde pública (1987) 21: 200-10.

Kashihara K, Maruyama T, Murota M, Nakahara Y. Positive Effects of Acute and Moderate Physical Exercise on Cognitive Function. J Physiol Anthropol (2009), 28: 155–164

Liu-Ambrose T, Donaldson MG. Exercise and cognition in older adults: is there a role for resistance training programmers? Br. J. Sports Med. (2009) 43: 25-27

Maia ALG , Godinho C , Ferreira ED , Almeida V,Schuh A, Kaye J, Chaves MLF. Aplicação da versão brasileira da escala de avaliação clínica da demëncia (clinical dementia Rating – CDR) em amostras de pacientes com demëncia. Arq Neuropsiquiatr (2006) 64(2-B): 485-489

McMorris T, Mielcarz G, Harris RC, Swain JP, Howard A. Creatine Supplementation and Cognitive Performance in Elderly Individuals. Aging, Neuropsychology, and Cognition (2007), 14: 517–528

Miyamoto ST, Lombardi Junior I, Berg KO, Ramos LR, Natour J. Brazilian version of the Berg balance scale. Braz J Med Biol Res (2004) 37: 1411-1421

Mota MMPE, Banhato EFC, Silva KCA, Cupertino APFB. Triagem cognitiva: comparações entre o mini-mental e o teste de trilhas Estudos de Psicologia (2008), Campinas 25(3): 353-359

Newcomer KL, Krug HE, Mahowald ML. Validity and reliability of the timed-stands test for patients with rheumatoid arthritis and other chronic diseases. J Rheumatol (1993) 20(1): 21-7

Ochiai ME, Franco LLS, Gebara OCE, Nussbacher A, Pierre JBSH, Rays J, Barreto ACP, Wajngarten M. Associação entre Evolução da Função Cognitiva e Mortalidade após a Alta Hospitalar em Pacientes Idosos com Insuficiência Cardíaca Avançada. Arq Bras Cardiol (2004) 82 (nº 3): 251-4

[Obrig H](http://www.ncbi.nlm.nih.gov/pubmed?term="Obrig H"%5BAuthor%5D), [Hirth C](http://www.ncbi.nlm.nih.gov/pubmed?term="Hirth C"%5BAuthor%5D), [Junge-Hülsing JG](http://www.ncbi.nlm.nih.gov/pubmed?term="Junge-Hülsing JG"%5BAuthor%5D), [Döge C](http://www.ncbi.nlm.nih.gov/pubmed?term="Döge C"%5BAuthor%5D), [Wolf T](http://www.ncbi.nlm.nih.gov/pubmed?term="Wolf T"%5BAuthor%5D), [Dirnagl U](http://www.ncbi.nlm.nih.gov/pubmed?term="Dirnagl U"%5BAuthor%5D), [Villringer A](http://www.ncbi.nlm.nih.gov/pubmed?term="Villringer A"%5BAuthor%5D). Cerebral oxygenation changes in response to motor stimulation. [J Appl Physiol.](javascript:AL_get(this, 'jour', 'J Appl Physiol.');) 1996 Sep;81(3):1174-83.

Papaléo MN. O estudo da Velhice no século XX: Histórico, Definição do Campo e Termos Básicos. In: Freitas, E.V. etal. Tratado de Geriatria e Gerontologia. Rio de Janeiro. Ed Guanabara. Pag. 2 a 12

Phillips WT, Batterham AM, Valenzuela JE, Burkett LN. Reliability of Maximal Strength Testing in Older Adults. Arch Phys Med Rehabil. 2004; 85.

Podsiadlo D, Richardson S. The timed ‘‘Up & Go’’: A test of basic functional mobility for frail elderly persons. J Am Geriatr Soc (1991) 39: 142–8

[Radosevich PM](http://www.ncbi.nlm.nih.gov/pubmed?term="Radosevich PM"%5BAuthor%5D), [Nash JA](http://www.ncbi.nlm.nih.gov/pubmed?term="Nash JA"%5BAuthor%5D), [Lacy DB](http://www.ncbi.nlm.nih.gov/pubmed?term="Lacy DB"%5BAuthor%5D), [O'Donovan C](http://www.ncbi.nlm.nih.gov/pubmed?term="O'Donovan C"%5BAuthor%5D), [Williams PE](http://www.ncbi.nlm.nih.gov/pubmed?term="Williams PE"%5BAuthor%5D), [Abumrad NN](http://www.ncbi.nlm.nih.gov/pubmed?term="Abumrad NN"%5BAuthor%5D). Effects of low- and high-intensity exercise on plasma and cerebrospinal fluid levels of ir-beta-endorphin, ACTH, cortisol, norepinephrine and glucose in the conscious dog. [Brain Res.](javascript:AL_get(this, 'jour', 'Brain Res.');) 1989 Sep 25;498(1):89-98.

[Rikli RE](http://www.ncbi.nlm.nih.gov/pubmed?term="Rikli RE"%5BAuthor%5D), [Edwards DJ](http://www.ncbi.nlm.nih.gov/pubmed?term="Edwards DJ"%5BAuthor%5D).Effects of a three-year exercise program on motor function and cognitive processing speed in older women. [Res Q Exerc Sport.](javascript:AL_get(this, 'jour', 'Res Q Exerc Sport.');) 1991 Mar;62(1):61-7.

Sachdev P. Homocisteína e transtornos psiquiátricos. Revista Brasileira de Psiquiatria. vol.26 no.1 Mar. 2004

Santos DL, Milano ME, Rosat R. Exercício Físico e Memória. Revista Paulista de Educação Física (1998) 12: 95-106

Scagliusi FB, Polacow VO, Artioli GG, Benatti FB, Lancha AH Jr. Selective underreporting of energy intake in women: magnitude, determinants, and effect of training. J Am Diet Assoc. 2003 Oct; 103 (10): 1306-13.

Sheik JI; YesavageJA. Geriatric Depression Scale: recent evidence and development of a shorter version. Clin. Geront. 1986; 5:165-72.

Shulman RG, Rothman DL, Behar KL, Hyder F. Energetic basis of brain activity implications for neuroimaging. Trends Neurosci (2004) 27: 489–495

Shumway-Cook A, Horak FB. Assessing the influence of sensory interaction on balance, suggestion from the field. Phys Ther (1986) 66: 1548-50

Silva LCA, Adda CC. Aspectos cognitivos relacionados à noção de intervalos de tempo. Bras Psiquiatr (2007) 56(2): 120-126

Spirduso WW. Physical fitness, aging, and psychomotor speed: a review. [J Gerontol.](javascript:AL_get(this, 'jour', 'J Gerontol.');) 1980 Nov;35(6):850-65.

Spirduso WW, Francis KL, MacRae PG. Physical Dimensions of Aging. Human Kinetics, 2^nd^ ed (2005)

Spreen O, Strauss E. Executive functions. In: Spreen O, Strauss E. A Compendium of Neuropsychological Tests. New York: Oxford University Press, 1998, p. 171-231.

Tinker A. The social implications of an ageing population. Mechanisms of Ageing and Development (2002) 123: 729–735

Umpierre D & Stein R. Hemodynamic and Vascular Effects of Resistance Training: Implications for Cardiovascular Disease. Arq Bras Cardiol 2007; 89(4) : 256-262

Watanabe A.; Kato N; Kato T. Effects of creatine on mental fatigue and cerebral hemoglobin oxygenation. Neurosci Res, v.42, n.4, p.279-285, 2002.

Wayne T. Phillips, PhD, FACSM, Alan M. Batterham, PhD, FACSM, Julie E. Valenzuela, MS, Lee N. Burkett, PhD. Reliability of Maximal Strength Testing in Older Adults. Arch Phys Med Rehabil Vol 85, February 2004

Wyss, M., et al. Creatine and creatinine metabolism. Physiol Rev (2000), v.80, n.3, Jul, p.1107-213
