# Supplementary material for: Creatine Supplementation Associated or Not with Strength Training upon Emotional and Cognitive Measures in Older Women: A Randomized Double-Blind Study
Source: PLoS One. 2013 Oct 3;8(10):e76301. doi: 10.1371/journal.pone.0076301 (PMC3789718; doi:10.1371/journal.pone.0076301)
Supplement: Protocol S2 — Trial Protocol (Portuguese). (DOC) [file pone.0076301.s003.doc]

**Universidade de São Paulo**

**Laboratório de Metabolismo Ósseo, Divisão de Reumatologia, Faculdade de Medicina**

**Laboratório de Avaliação e Condicionamento em Reumatologia, Divisão de Reumatologia, Faculdade de Medicina**

**Laboratório e Nutrição e Metabolismo Aplicados, Escola de Educação Física e Esporte**

**EFEITOS DO TREINAMENTO DE FORÇA ACOMPANHADO DA SUPLEMENTAÇÃO DE CREATINA NA FUNÇÃO COGNITIVA DE IDOSOS**

**Pesquisador Responsável:**

Profª Drª Rosa Maria Rodrigues Pereira

**Pesquisador Executante:**

Christiano Robles Rodrigues Alves

Prof. Dr. Bruno Gualano

São Paulo

EFEITOS DO TREINAMENTO DE FORÇA ACOMPANHADO DA SUPLEMENTAÇÃO DE CREATINA NA FUNÇÃO COGNITIVA DE IDOSOS

A literatura apresenta evidencias que destacam o papel protetor do exercício físico no declínio das funções cognitivas, sendo que as maiores perspectivas se relacionam ao exercício físico de predominância aeróbia. Entretanto, alguns pesquisadores também indicam a influência benéfica do exercício físico resistido nessa função e sugerem possíveis mecanismos para que ela ocorra. Outra estratégia não farmacológica proposta com o objetivo de abrandar as perdas cognitivas é a suplementação de creatina, pois essa amina parece apresentar um importante papel terapêutico e alguns autores indicam a sua influência no sistema nervoso central. Porém, nenhum estudo até o presente momento investigou a ação do treinamento físico resistido combinado com suplementação de creatina na função cognitiva de idosos. Sendo assim, o objetivo desse trabalho será avaliar uma possível sinergia entre ambas as estratégias. Para isso realizaremos um estudo duplo-cego, randomizado e controlado por placebo. Os sujeitos serão divididos em quatro grupos: Treinamento + Placebo (TR+P), Treinamento + Creatina (TR+Cr), Placebo (P) e Creatina (Cr). Durante 24 semanas os indivíduos dos grupos TR+P e TR+Cr serão submetidos a treinamento físico, enquanto os grupos P e Cr serão orientados a não ingressarem em nenhum programa de atividade física. Nos períodos pré, após 12 semanas e após 24 semanas de intervenção, todos os grupos realizarão testes de força muscular, avaliação nutricional, testes cognitivos.

**1. Introdução e Justificativa**

O processo de envelhecimento pode ser explicado como o somatório de alterações orgânicas, funcionais e psicológicas no ser humano. Essas alterações são inevitáveis e, ainda, podem ser agravadas por inúmeras doenças no decorrer da vida (Papaléo, 2002). A memória, linguagem, capacidade de associação, comparação, atenção e síntese merecem uma atenção especial por estarem diretamente ligadas à qualidade de vida da população idosa. No processo de envelhecimento ocorre um decréscimo na capacidade de evocação de novas informações, na manipulação das mesmas, na capacidade de realização de múltiplas tarefas simultaneamente, enfim, as funções executivas são afetadas de um modo geral durante o envelhecimento em comparação a adultos jovens (Spirduso *et al*, 2005).

Diante do aumento demográfico mundial da população idosa, alguns pesquisadores têm investigado possíveis estratégias para atenuar a perda das funções cognitivas. Dentre as não farmacológicas destacam-se o treinamento físico aeróbio (Blackwood *et al*, 1998; Kashihara *et al*, 2009; Davranche *et al*, 2009) e o de força (Liu-Ambrose *et al*, 2009), além da suplementação de creatina (McMorris *et al*, 2007; Andres *et al*, 2008; Bender, 2006).

Os estudos que visam investigar os efeitos do exercício aeróbio na função cognitiva não são recentes. Desde 1980 já havia publicações sobre a sua influência na cognição. Embora ainda haja uma grande discussão sobre o tema, principalmente quanto à intensidade com que esses exercícios devem ser realizados (Kashihara *et al*, 2009), alguns estudos com exercício aeróbio moderado (60% do VOmax) demonstram, através de espectroscopia por infra-vermelho, que o efeito é positivo na cognição, pois melhora o fluxo sanguíneo cerebral regional e, conseqüentemente, isso leva a uma melhor oferta de oxigênio e glicose na área do cérebro que controla as funções cognitivas (Obrig *et al*, 1996). Além disso, o exercício aeróbio agudo pode alterar os níveis dos neurotransmissores no Sistema Nervoso Central, como a acetilcolina, a dopamina, a noradrenalina, a adrenalina, o hormônio adrenocorticotrófico (ACTH) e a vasopressina, podendo ativar novamente a função cognitiva (Spirduso, 1980; Radosevich *et al*, 1989; Rikli *et al*, 1991). Maiores detalhes podem ser encontrados em um recente artigo (2009) de Kashihara *et al.*

O treinamento de força proporciona diversas adaptações metabólicas e hemodinâmicas no organismo, tais como a redução da gordura abdominal, melhoras nas concentrações de triglicérides no plasma, aumento do *High Density Lipoprotein-Colesterol* (HDL-C) e controle glicêmico (Guttierres APM & Martins JCB, 2008). Outras importantes adaptações são as mudanças na taxa de eliminação de glicose, aumento na competência de estocar glicogênio, aumento dos receptores GLUT 4 no músculo, aumento da sensibilidade a insulina e normalização da tolerância a glicose. Ainda é importante salientar os benefícios hemodinâmicos que o treinamento de força acarreta como a tendência em reduzir a pressão arterial sistólica e diastólica, promovendo a secreção de substâncias vasodilatadoras e a participação como componente valioso para a angiogênese, ou seja, formação de novos vasos capilares. Umpierre & Stein comentam da aparente eficácia do treinamento de força sobre a manutenção do fluxo sangüíneo periférico em indivíduos idosos, o que pode colaborar para minimizar a limitação funcional presente no envelhecimento ou em diferentes condições patológicas. Além disso, o treinamento de força aumenta a massa muscular e a força muscular máxima, podendo também atenuar no avanço da sarcopenia (Umpierre & Stein, 2007).

Recentemente, tem-se demonstrado que o treinamento de força também possui papel essencial sobre função cognitiva. Cassilhas *et at* (2007) demonstraram, através das baterias de testes *Wechsler Adult Intelligence Scale III (WAIS III), Wechsler Memory Scale-Revised (WSM-R),Toulouse-Pieron's concentration attention tes*t que 24 semanas de treinamento de força de duas diferentes intensidades, 50% e 80% de 1RM em duas séries de oito repetições cada, podem melhorar o desempenho cognitivo em homens idosos. Além disso, os pesquisadores encontraram um aumento nas concentrações séricas de Insulin-like Growth Factor (IGF-I) nos grupos treinados em força. Sabe-se que o aumento de IGF-I diminui a concentração plasmática de homocisteína, que é um aminoácido sulfuroso derivado da desmetilação da metionina que em altos níveis pode causar lesões cerebrais e transtornos neuropsiquiátricos (Liu-Ambrose *et at*, 2009; Ducloux *et at*, 2002). A homocisteína é pró-aterogênica e pró-trombótica, aumentando o risco de acidente vascular cerebral e podendo ter um efeito neurotóxico direto (Sachdev P, 2004). O IGF-I também modula os níveis de acetilcolina no hipocampo que exercem importantes funções no desenvolvimento do Sistema Nervoso Central (Arwert *et at*, 2005). Em outro estudo, Perrig-Chiello et al evidenciam que 8 semanas de treinamento de força melhorou significativamente a auto-atenção, pensamentos egocêntricos, recordação, memória e ansiedade em indivíduos idosos. Em uma recente revisão (2009), Liu-Ambrose *et at* comentam sobre a necessidade de mais pesquisas para definir claramente a função do exercício resistido na prevenção do declínio cognitivo que afeta a população idosa.

Outra estratégia supostamente capaz de abrandar a perda da cognição é a suplementação de creatina. Esta amina sintetizada pelo fígado, rins e pâncreas ou obtida via alimentação, encontra-se primordialmente (95%) no músculo esquelético, sendo o restante distribuído no intestino, músculo liso, testículos e no cérebro (Wyss *et at*, 2000). A creatina é encontrada no corpo humano nas formas livre (60 a 70%) e fosforilada (30 a 40%). Um dos principais papéis do sistema PCr-CK é a transferência de energia da mitocôndria para o citosol, especialmente em tecidos cuja demanda energética é elevada, como o músculo e cérebro (Gualano et al, 2009).

O cérebro é responsável por cerca de 20% do consumo energético total do organismo (Shulman *et at*, 2004). Recentemente, constatou-se que a creatina oralmente administrada é capaz de ultrapassar a barreira hematoencefálica e elevar as concentrações cerebrais desse substrato (Andres et al, 2008). Em uma recente revisão de literatura, Andres et al destacam que o sistema fosforilcreatina (PCR) desempenha um papel fundamental no metabolismo energético do cérebro devido a ressíntese de trifosfato de adenosina (ATP) pela creatine quinase (CK). A consolidação da memória, por exemplo, é realizada no hipocampo e requer muita energia dependente da hidrólise de ATP (Mcmorris et al, 2007). Além disso, os resultados de alguns estudos indicam que a suplementação de creatina é capaz de melhorar a função cognitiva em indivíduos saudáveis jovens (Watanabe et al. 2002) e idosos (Mcmorris et al., 2007). Além da importante função de fornecimento de energia para o cérebro proposta para explicar os melhores desempenhos em cálculos matemáticos de adultos jovens (24.3 +/- 9.1 anos), Watanabe et al demonstram, por meio de espectroscopia no infra-vermelho, que a suplementação de creatina, assim como o exercício físico (Kashihara *et al*, 2009), parece também ser capaz de aumentar a oxigenação cerebral (Watanabe et al, 2002). Entretanto, os mecanismos pelos quais esse aumento de oxigenação atuaria sobre a cognição ainda foram pouco explorados.

Sabe-se que idosos necessitam de mais energia para a realização de tarefas cognitivas quando comparados a indivíduos mais jovens (Behzadi & Liu, 2005; Toescu, 2005). Embora estudos utilizando espectroscopia por ressonância magnética demonstrem que o nível de creatina cerebral tende a aumentar com a idade, tal incremento não é o suficiente para compensar as perdas cognitivas, levantando a possibilidade de a suplementação de creatina atenuar tal quadro (McMorris T *et al*, 2007). Além disso, Ellis e Rosenfeld (2004) demonstraram que a creatina é capaz de aumentar a ação antioxidante e McMorris *et al*, 2007 comentam sobre um aumento das concentrações de dopamina cerebral, que é um neurotransmissor essencial nas tarefas cognitivas, conforme previamente discutido. Em modelos *in vitro*, têm sido demonstrados efeitos benéficos da creatina sobre a recaptação de glutamato, que é reconhecidamente neurotóxico em altas concentrações (Andres RH *et al*, 2008). Considerando-se esses dados em conjunto, é possível afirmar que a suplementação de creatina exerce um papel importante e promissor na função cognitiva.

Tendo em vista os benefícios promovidos pelo treinamento de força bem como da suplementação de creatina sobre a cognição, será objetivo investigar os efeitos combinados de ambas as estratégias sobre a função cognitiva de idosos.

**2.** **Objetivos**

**2.1 Objetivo Geral**

Investigar os efeitos do treinamento de força combinado com a suplementação de creatina na função cognitiva de idosos.

- 1. **Objetivos Específicos**

Avaliar o desempenho em testes cognitivos e testes de força nos períodos pré, após 12 semanas e após 24 semanas.

**3. Metodologia**

- 1. **Protocolo Experimental**

Será conduzido um estudo clinico randomizado, duplo-cego e controlado por placebo, onde todos os sujeitos passarão por avaliação médica com o intuito de verificar os critérios de elegibilidade. Serão 60 voluntários divididos aleatoriamente em 4 grupos: Treinamento + Placebo (TR+P), Treinamento + Creatina (TR+Cr), Placebo (P) e Creatina (Cr).

Ao longo de 24 semanas, os sujeitos dos grupos TR+P e TR+Cr serão submetidos a treinamento de força (ver item 3.6), enquanto os sujeitos dos grupos P e Cr serão orientados a não entrar em nenhum programa de atividade física. No período pré-intervenção (PRÉ), após 12 semanas (PÓS 12) e após 24 semanas (PÓS 24) os voluntários realizarão os testes cognitivos, testes de força e coletas sanguíneas para mensurar as variáveis desejadas.

- 1. **Amostra**

Serão selecionados 60 voluntários que passarem por entrevistas e avaliação médica (incluindo teste ergométrico) para análise do enquadramento nos critérios de inclusão do estudo, sendo que todos assinarão o termo de consentimento livre e esclarecido, conforme as normas do Comitê de Ética.

Critérios de Inclusão:

- Idosos: 60 a 80 anos
- Sexo feminino
- Não praticante de nenhum treinamento físico a pelo menos um ano.
- Não suplementado com creatina a pelo menos 6 meses.

Critérios de Exclusão:

- Indivíduos que não freqüentaram a escola e incapazes de ler uma frase simples.
- Acometimentos no aparelho locomotor ou doenças cardiovasculares que sejam limitantes para a prática dos exercícios
- Taxa de filtragem glomerular menor que 30ml/kg/min
- Fumantes
  1. **Teste Ergométrico**

Os voluntários receberão, por escrito, todas as instruções relevantes à execução do teste. Durante o teste, serão verificadas as respostas pressóricas, por meio de esfigmomanômetro de coluna de mercúrio; cronotrópicas e eletrocardiográficas, por meio de eletrocardiograma; percepção subjetiva de esforço, com auxílio da escala de Borg. Todos os testes serão conduzidos sob a supervisão de um médico cardiologista.

- 1. **Avaliação Nutricional**

Os participantes serão orientados a não alterar seu consumo alimentar ao longo do estudo. Para avaliar possíveis alterações serão realizadas avaliações nutricionais nos períodos PRÉ e PÓS 12. A avaliação consistirá da análise do diário alimentar de três dias, sendo dois dias úteis da semana e um dia do final de semana (Scagliusi F, 2003).

- 1. **Suplementação**

A suplementação será realizada conforme o modelo duplo-cego, ou seja, nem os pesquisadores, nem os voluntários terão pronto conhecimento se estão ingerindo creatina ou placebo. Uma nutricionista será responsável pela randomização dos voluntários nos dois grupos e denominação de acordo com sua própria escolha em: Grupo 1 e Grupo 2. Os grupos serão revelados apenas após a análise estatística dos dados.

Os voluntários receberão durante os cinco primeiros dias 20g de suplemento por dia divididos em quatro sacos plásticos do tipo “zip lock” dentro de uma sacola devidamente identificada com o seu nome. A partir do sexto dia cada paciente consumirá o conteúdo de 1 sacos por dia (5 g). Todos os voluntários serão orientados a ingerir a suplementação acompanhada de suco, preferencialmente durante uma refeição. A suplementação não deve ser consumida com cafeína.

Será fornecida a cada paciente uma tabela mensal onde deverá constar o horário em que o suplemento foi ingerido, o acompanhamento (suco de laranja, por exemplo) e uma etiqueta correspondente ao saco “zip-lok”. Cada saco plástico será rotulado com uma etiqueta denominada: Grupo 1 ou Grupo 2.

Este método é uma adaptação de um estudo prévio (Gualano et al. 2008), onde os relatos indicam uma aderência de 100% ao protocolo.

- 1. **Avaliação da Força e Função Muscular**

A força muscular será determinada através do teste de uma repetição máxima (1RM). O teste será realizado em dois equipamentos diferentes, sendo eles: *chest press* e *leg press.* Haverá um aquecimento específico de 8 repetições com uma carga de aproximadamente 50% e, após 2 minutos de descanso, 3 repetições com aproximadamente 70% do 1RM estimado para o indivíduo. Após 3 minutos do aquecimento específico, o individuo deve realizar 5 tentativas de uma repetição com 3 minutos de descanso entre elas. Para evitar a influência de adaptações neurais e aprendizagem motora do movimento serão realizadas três sessões de familiarização já relatadas na literatura como suficientes para ocorrer uma estabilização da medida (Phillips et al, 2004).

Para complementar a avaliação da força muscular serão efetuados testes de contração isométrica (pressão palmar e tração lombar) com o auxilio de dinamômetros. Esses testes devem ser feitos duas vezes e em dias diferentes (48 horas de descanso) onde, em cada dia, devem ser cumpridas 3 tentativas para cada dinamômetro. A força de preensão palmar é mensurada com o braço dominante do individuo estendido ao lado do corpo e na tração lombar o individuo deverá posicionar-se em pé sobre a plataforma do dinamômetro com os joelhos estendidos e o tronco flexionado à frente em um ângulo de aproximadamente 120º, a partir dessa posição o individuo deve aplicar força para tentar retornar a posição “ereta” (Brown, 2003).

Os testes funcionais têm o objetivo de avaliar o ganho de força para atividades do cotidiano. Serão dois testes diferentes aplicados duas vezes cada e novamente em dias diferentes. O primeiro teste funcional é uma adaptação do teste *“Timed-stands test”* (Newcomer *et al*, 1993), onde, no nosso caso, ele vai consistir do número de vezes que o individuo consegue se levantar e sentar novamente em uma cadeira formando um ângulo de aproximadamente 90 graus no joelho, durante 30 segundos. O segundo teste é denominado *“Timed up-and-go”* (Podsiadlo *et al*, 1991) onde o paciente deverá se levantar de uma cadeira, andar 3 metros a frente e retornar para a cadeira. O tempo total gasto será aferido.

- 1. **Treinamento de Força**

O treinamento de força consiste de duas sessões semanais durante as 24 semanas do estudo. Uma sessão completa de treinamento (aquecimento, exercícios de força e alongamentos) terá a duração de cerca de 50 minutos, sendo importante destacar que para avaliar a aderência dos voluntários será controlada rigorosamente a presença dos mesmos.

Em cada sessão haverá um aquecimento geral em esteira, cerca de 5 minutos, seguido por um aquecimento específico (50% do 1RM) antes de cada exercício. Após o aquecimento, devem ser realizadas 3 séries de 8 a 12 repetições máximas para oito exercícios diferentes, sempre respeitando um descanso de 90 segundos entre as séries.

Os sete exercícios foram protocolados com o objetivo de trabalhar os grandes grupamentos musculares. A princípio uma sessão de treinamento será composta pelos seguintes exercícios: Supino no Banco*, Leg Press,* Remada, Cadeira Extensora, Puxador, Meio Agachamento Livre e Reto Abdominal (flexão de quadril). Após a realização dos exercícios, serão realizados alongamentos de intensidade leve a moderada para os principais grupamentos musculares.

- 1. **Testes Cognitivos**

A avaliação cognitiva será mensurada através dos testes cognitivos: MEEM, Teste de Trilhas (formas A e B), teste de Stroop, Lista de Palavras de GERAD e a Escala de Depressão Geriátrica Abreviada.

O MEEM é composto por itens que avaliam orientação, espaço temporal, memória imediata, evocação de memória, atenção e linguagem, com um escore total de 30 pontos (Folstein et al., 1975; Brucki et al., 2003). Além disso, usaremos o Teste de Trilhas A e B. O Teste de Trilha A consiste em ligar, em ordem crescente e por meio de uma linha contínua, todos os números (1,2,3...) formando uma figura. O Teste de Trilha B é solicitado ao examinando que ligue alternadamente número e letra (1-A, 2-B, 3-C...). Ambos têm como objetivo avaliar a atenção, seqüenciamento, flexibilidade mental, busca visual e função motora, sendo que o Teste de Trilha B ainda exige maior capacidade de atenção e habilidade para fazer mudanças conceituais alternadas (Mota *et al*, 2008). Para avaliar atenção seletiva, velocidade de processamento, alternância de tarefas, capacidade de inibição de estímulos irrelevantes e de inibição de respostas será utilizado o teste de Stroop (Spreen & Strauss, 1998). Para avaliar a memória será usada a lista de palavras do CERAD (Bertolucci et al., 2001). Ainda será aplicado a Escala de Depressão Geriátrica Abreviada **(**Sheik et al, 1986) para a avaliação da depressão.

- 1. **Avaliação da composição corporal**

A avaliação da composição corporal (massa magra, massa gorda e conteúdo mineral ósseo) será feita por DXA utilizando o aparelho Hologic Discovery, pré e após 24 semanas da intervenção.

- 1. **Locais de execução dos procedimentos**

O treinamento físico, a avaliação nutricional, o teste ergométrico e a aplicação dos testes cognitivos serão conduzidos no Laboratório de Avaliação e Condicionamento em Reumatologia no Hospital das Clínicas (LACRE – HCFMUSP). A avaliação da composição corporal será realizada no Laboratório de Metabolismo Ósseo – Reumatologia (LIM-17) da FMUSP.

- 1. **Análises estatísticas**

Os dados serão expressos em média ± desvio padrão ou porcentagem. As diferenças (final - inicial) entre os quatro grupos serão analisadas por ANOVA, seguida do test de Tukey ou teste Kruskal-Wallis (não paramétrico). O teste qui-quadrado ou exato de Fisher será utilizado para comparar as variáveis categóricas. O nível de significância adotado para rejeitar a hipótese nula será de P ≤ 0,05.

**4. Cronograma de Execução**

Após a aprovação do comitê de ética a duração total do projeto será de 24 semanas. De acordo com essa distribuição:

- Recrutamento e Seleção de Voluntários – 8 semanas
- Coletas e Testes PRÉ – 8 semanas
- Intervenção – 12 semanas
- Coletas e Testes PÓS 12 semanas
- Intervenção – 12 semanas
- Coletas e Testes PÓS 24 semanas
- Análise e publicação dos dados – 8 semanas

**5. Alterações do projeto inicial**

As seguintes alterações se fizeram necessárias em decorrência de problemas técnicos e ou de ajustes metodológicos julgados necessários.

1) Inclusão de testes cognitivos adicionais, incluído um questionário específico para cognição.

2) Exclusão do exame DXA e testes de função muscular.

Todas as alterações estão de acordo com os princípios éticos preconizados pelo Conselho Nacional de Ética em Pesquisa (CONEP).

**6. Referências Bibliográficas**

Andres RH, Ducray AD, Schlattner U, Wallimann T, Widmer HR. Functions and effects of creatine in the central nervous system. Brain Research Bulletin (2008) 76: 329–34

Arwert LI, Deijen JB, Drent ML. The relation between insulin-like growth factor I levels and cognition in healthy elderly: A meta-analysis. Growth Hormone & IGF Research (2005) 15:416–422

Atalaia-Silva KC, Lourenço RA. Tradução, adaptação e validação de construto do Teste do Relógio aplicado entre idosos no Brasil. Rev Saúde Pública (2008) 42(5): 930-7

Bender A, Koch W, Elstner M, Schombacher Y, Bender J, Moeschl M, Gekeler F, Muller-Myhsok B, Gasser T, Tatsch K, Klopstock T. Creatine supplementation in Parkinson disease: a placebo-controlled randomized pilot trial. Neurology (2006) 67: 1262–1264

Berg KO, Wood-Dauphinee SL, Williams JI, Gayton D. Measuring balance in the elderly: Preliminary development of an instrument. Physiotherapy Canada (1989) 41: 304-11

Blackwood SK, MacHale SM, Power MJ, Goodwin GM, Lawrie SM. Effects of exercise on cognitive and motor function in chronic fatigue syndrome and depression. J Neurol Neurosurg Psychiatry (1998) 65: 541–546

Brucki SMD, Nitrini R, Caramelli P, Bertolucci PHF, Okamoto IH. Sugestões para o uso do Mini-exame de Estado Mental no Brasil. Arq Neuropsiquiatr 2003;61(3-B):777-781

Bustamante SEZ , Bottino CMC, Lopes MA, Azevedo D, Hototian SR , Litvoc J, Jacob Filho W. Instrumentos combinados na avaliação de demência em idosos. Arq Neuropsiquiatr (2003) 61: 601-606

Brown LE & WEIR JP. ASEP Procedures Recommendation I: Accurate Assessment Of Muscular Strength And Power. JEPonline. 2001;4(3):1-21.

Cassilhas RC, Viana VA, Grassmann V, [Santos RT](http://www.ncbi.nlm.nih.gov/pubmed?term="Santos RT"%5BAuthor%5D&itool=EntrezSystem2.PEntrez.Pubmed.Pubmed_ResultsPanel.Pubmed_RVAbstract), [Santos RF](http://www.ncbi.nlm.nih.gov/pubmed?term="Santos RF"%5BAuthor%5D&itool=EntrezSystem2.PEntrez.Pubmed.Pubmed_ResultsPanel.Pubmed_RVAbstract), [Tufik S](http://www.ncbi.nlm.nih.gov/pubmed?term="Tufik S"%5BAuthor%5D&itool=EntrezSystem2.PEntrez.Pubmed.Pubmed_ResultsPanel.Pubmed_RVAbstract), [Mello MT](http://www.ncbi.nlm.nih.gov/pubmed?term="Mello MT"%5BAuthor%5D&itool=EntrezSystem2.PEntrez.Pubmed.Pubmed_ResultsPanel.Pubmed_RVAbstract). The impact of resistance exercise on the cognitive function of the elderly. Med Sci Sports Exerc (2007) 39:1401–7

Davranche K, McMorris T. Specific effects of acute moderate exercise on cognitive control. Brain and Cognition (2009) 69: 565–570

Ducloux D, Motte G, Nguyen NU, Abdelfatah A, Gibey R, Chalopin JM. Homocysteine, nutritional status and insulin in renal transplant recipients. Nephrol Dial Transplant (2002) 17: 1674–1677

Fava DC, Kristensen CH, Melo WV, Araújo LB. Construção e validação de tarefa de Stroop Emocional para avaliação de viés de atenção em mulheres com Transtorno de Ansiedade Generalizada. Paidéia (2009) 19 (43): 159-165

Fernandes RCL, Silva KS, Bonan C, Zaha SEV, Marinheiro LPF. Avaliação da cognição de mulheres no climatério com o Mini-Exame do Estado Mental e o Teste de Memória da Lista de Palavras. Cad. Saúde Pública (2009), Rio de Janeiro, 25 (9): 1883-1893

Folstein MF, Folstein SE, McHugh PR. Mini-Mental State: a practical method for grading the cognitive state of patients for clinician. J Psychiatr Res 1975;12:189-198.

Gomes CMA, Borges O. Qualidades Psicométricas de um conjunto de 45 testes. Fractal Revista de Psicologia (2008) 20: 195-208

Gualano B, Artioli GG, Poortmans JR, Lancha AH Jr. Exploring the therapeutic role of creatine supplementation. Amino Acids (2009)

Gualano B, Novaes RB, Artioli GG, Freire TO, Coelho DF, Scagliusi FB, Rogeri PS, Roschel H, Ugrinowitsch C, Lancha AH Jr. Effects of creatine supplementation on glucose tolerance and insulin sensitivity in sedentary healthy males undergoing aerobic training. Amino Acids (2008) 34: 245–250

Guttierres APM & Martins JCB. Effects of Resistance Training Over Metabolic Sydrome Risk Factors. Rev Bras Epidemiol. 2008; 11(1): 147-58

Kalache A, Veras RP, Ramos LR. O envelhecimento da população mundial: um desafio novo. Revista Saúde pública (1987) 21: 200-10.

Kashihara K, Maruyama T, Murota M, Nakahara Y. Positive Effects of Acute and Moderate Physical Exercise on Cognitive Function. *J Physiol Anthropol (2009), 28: 155–164*

Liu-Ambrose T, Donaldson MG. Exercise and cognition in older adults: is there a role for resistance training programmers? *Br. J. Sports Med. (*2009) 43: 25-27

Maia ALG , Godinho C , Ferreira ED , Almeida V,Schuh A, Kaye J, Chaves MLF. Aplicação da versão brasileira da escala de avaliação clínica da demëncia (clinical dementia Rating – CDR) em amostras de pacientes com demëncia. Arq Neuropsiquiatr (2006) 64(2-B): 485-489

McMorris T, Mielcarz G, Harris RC, Swain JP, Howard A. Creatine Supplementation and Cognitive Performance in Elderly Individuals. Aging, Neuropsychology, and Cognition (2007), 14: 517–528

Miyamoto ST, Lombardi Junior I, Berg KO, Ramos LR, Natour J. Brazilian version of the Berg balance scale. Braz J Med Biol Res (2004) 37: 1411-1421

Mota MMPE, Banhato EFC, Silva KCA, Cupertino APFB. Triagem cognitiva: comparações entre o mini-mental e o teste de trilhas Estudos de Psicologia (2008), Campinas 25(3): 353-359

Newcomer KL, Krug HE, Mahowald ML. Validity and reliability of the timed-stands test for patients with rheumatoid arthritis and other chronic diseases. J Rheumatol (1993) 20(1): 21-7

Ochiai ME, Franco LLS, Gebara OCE, Nussbacher A, Pierre JBSH, Rays J, Barreto ACP, Wajngarten M. Associação entre Evolução da Função Cognitiva e Mortalidade após a Alta Hospitalar em Pacientes Idosos com Insuficiência Cardíaca Avançada. Arq Bras Cardiol (2004) 82 (nº 3): 251-4

[Obrig H](http://www.ncbi.nlm.nih.gov/pubmed?term="Obrig H"%5BAuthor%5D), [Hirth C](http://www.ncbi.nlm.nih.gov/pubmed?term="Hirth C"%5BAuthor%5D), [Junge-Hülsing JG](http://www.ncbi.nlm.nih.gov/pubmed?term="Junge-Hülsing JG"%5BAuthor%5D), [Döge C](http://www.ncbi.nlm.nih.gov/pubmed?term="Döge C"%5BAuthor%5D), [Wolf T](http://www.ncbi.nlm.nih.gov/pubmed?term="Wolf T"%5BAuthor%5D), [Dirnagl U](http://www.ncbi.nlm.nih.gov/pubmed?term="Dirnagl U"%5BAuthor%5D), [Villringer A](http://www.ncbi.nlm.nih.gov/pubmed?term="Villringer A"%5BAuthor%5D). Cerebral oxygenation changes in response to motor stimulation. [J Appl Physiol.](javascript:AL_get(this, 'jour', 'J Appl Physiol.');) 1996 Sep;81(3):1174-83.

Papaléo MN. O estudo da Velhice no século XX: Histórico, Definição do Campo e Termos Básicos. In: Freitas, E.V. etal. Tratado de Geriatria e Gerontologia. Rio de Janeiro. Ed Guanabara. Pag. 2 a 12

Phillips WT, Batterham AM, Valenzuela JE, Burkett LN. Reliability of Maximal Strength Testing in Older Adults. Arch Phys Med Rehabil. 2004; 85.

Podsiadlo D, Richardson S. The timed ‘‘Up & Go’’: A test of basic functional mobility for frail elderly persons. J Am Geriatr Soc (1991) 39: 142–8

[Radosevich PM](http://www.ncbi.nlm.nih.gov/pubmed?term="Radosevich PM"%5BAuthor%5D), [Nash JA](http://www.ncbi.nlm.nih.gov/pubmed?term="Nash JA"%5BAuthor%5D), [Lacy DB](http://www.ncbi.nlm.nih.gov/pubmed?term="Lacy DB"%5BAuthor%5D), [O'Donovan C](http://www.ncbi.nlm.nih.gov/pubmed?term="O'Donovan C"%5BAuthor%5D), [Williams PE](http://www.ncbi.nlm.nih.gov/pubmed?term="Williams PE"%5BAuthor%5D), [Abumrad NN](http://www.ncbi.nlm.nih.gov/pubmed?term="Abumrad NN"%5BAuthor%5D). Effects of low- and high-intensity exercise on plasma and cerebrospinal fluid levels of ir-beta-endorphin, ACTH, cortisol, norepinephrine and glucose in the conscious dog. [Brain Res.](javascript:AL_get(this, 'jour', 'Brain Res.');) 1989 Sep 25;498(1):89-98.

[Rikli RE](http://www.ncbi.nlm.nih.gov/pubmed?term="Rikli RE"%5BAuthor%5D), [Edwards DJ](http://www.ncbi.nlm.nih.gov/pubmed?term="Edwards DJ"%5BAuthor%5D).Effects of a three-year exercise program on motor function and cognitive processing speed in older women. [Res Q Exerc Sport.](javascript:AL_get(this, 'jour', 'Res Q Exerc Sport.');) 1991 Mar;62(1):61-7.

Sachdev P. Homocisteína e transtornos psiquiátricos**.** Revista Brasileira de Psiquiatria. vol.26 no.1 Mar. 2004

Santos DL, Milano ME, Rosat R. Exercício Físico e Memória. Revista Paulista de Educação Física (1998) 12: 95-106

Scagliusi FB, Polacow VO, Artioli GG, Benatti FB, Lancha AH Jr. Selective underreporting of energy intake in women: magnitude, determinants, and effect of training. J Am Diet Assoc. 2003 Oct; 103 (10): 1306-13.

Sheik JI; YesavageJA. Geriatric Depression Scale: recent evidence and development of a shorter version. Clin. Geront. 1986; 5:165-72.

Shulman RG, Rothman DL, Behar KL, Hyder F. Energetic basis of brain activity implications for neuroimaging. Trends Neurosci (2004) 27: 489–495

Shumway-Cook A, Horak FB. Assessing the influence of sensory interaction on balance, suggestion from the field. Phys Ther (1986) 66: 1548-50

Silva LCA, Adda CC. Aspectos cognitivos relacionados à noção de intervalos de tempo. Bras Psiquiatr (2007) 56(2): 120-126

Spirduso WW. Physical fitness, aging, and psychomotor speed: a review. [J Gerontol.](javascript:AL_get(this, 'jour', 'J Gerontol.');) 1980 Nov;35(6):850-65.

Spirduso WW, Francis KL, MacRae PG. Physical Dimensions of Aging. Human Kinetics, 2^nd^ ed (2005)

Spreen O, Strauss E. Executive functions. In: Spreen O, Strauss E. A Compendium of Neuropsychological Tests. New York: Oxford University Press, 1998, p. 171-231.

Tinker A. The social implications of an ageing population. Mechanisms of Ageing and Development (2002) 123: 729–735

Umpierre D & Stein R. Hemodynamic and Vascular Effects of Resistance Training: Implications for Cardiovascular Disease. Arq Bras Cardiol 2007; 89(4) : 256-262

Watanabe A.; Kato N; Kato T. Effects of creatine on mental fatigue and cerebral hemoglobin oxygenation. Neurosci Res, v.42, n.4, p.279-285, 2002.

Wayne T. Phillips, PhD, FACSM, Alan M. Batterham, PhD, FACSM, Julie E. Valenzuela, MS, Lee N. Burkett, PhD. Reliability of Maximal Strength Testing in Older Adults. Arch Phys Med Rehabil Vol 85, February 2004

Wyss, M.*, et al.* Creatine and creatinine metabolism. Physiol Rev (2000), v.80, n.3, Jul, p.1107-213
